# Sleep is a therapeutic window for photostimulation of drainage of aging brain

**DOI:** 10.1007/s12200-025-00168-0

**Published:** 2025-11-19

**Authors:** Terskov Andrey, Adushkina Viktoria, Shirokov Alexander, Navolokin Nikita, Blokhina Inna, Zlatogorskaya Daria, Semiachkina-Glushkovskaia Anastasiia, Konstancia Sonina, Evsyukova Arina, Elizarova Inna, Tuzhilkin Matvey, Dmitrenko Alexander, Dubrovsky Alexander, Myagkov Dmitry, Popov Sergey, Tuktarov Dmitry, Ilyukov Egor, Tzoy Maria, Fedosov Ivan, Semyachkina-Glushkovskaya Oxana

**Affiliations:** 1 Department of Biology, Saratov State University, Saratov, 410012 Russia; 2 Institute of Biochemistry and Physiology of Plants and Microorganisms, Russian Academy of Sciences, Saratov, 410049 Russia; 3 Department of Pathological Anatomy, Saratov State Medical University, Saratov, 410012 Russia; 4 Department of Computer Science and Information Technology, Saratov State University, Saratov, 410012 Russia; 5 Institute of Physics, Saratov State University, Saratov, 410012 Russia; 6 MOE Key Laboratory for Biomedical Photonics, Wuhan National Laboratory for Optoelectronics-Advanced Biomedical Imaging Facility, Huazhong University of Science and Technology, Wuhan, 430074 China

**Keywords:** Photobiomodulation, Aging brain, Meningeal lymphatic vessels, Sleep, Brain drainage, Cognitive function

## Abstract

Age is a limiting factor in the efficacy of photobiomodulation (PBM) for brain drainage and cognitive functions. Meningeal lymphatic vessels (MLVs) are “tunnels” for removal of toxins from the brain and the target of PBM. Age-related decline in the MLV functions is one of the mechanisms by which the effects of PBM on brain drainage and cognitive process are limited. Sleep is a time of natural activation of brain drainage. Recent findings have shown that PBM during sleep has greater effects on lymphatic clearance of beta-amyloid and cognitive function in young and middle-age mice. Based on these data, this study tested the hypothesis that sleep enhances the effects of PBM on MLVs and cognitive function in the aging brain. Indeed, the results revealed that PBM during sleep, but not during wakefulness, has stimulatory effects on lymphatic clearance of beta-amyloid from the brain of old mice that improves memory. In sleep deficit experiments, it was found that chronic sleep deprivation is accompanied by suppression of brain drainage and removal of metabolites from the brain, such as beta-amyloid, tau, glutamate, lactate and glucose in young, middle-aged and most significantly in old mice. The course of PBM during sleep contributed better than in wakefulness to the restoration of the brain level of tested metabolites in young and middle-aged mice, while in old mice only PBM during sleep was effective. These results open a new strategy for the use of PBM during sleep to improve the efficacy of PBM on clearance of toxic metabolites from the brain, especially in aged subjects in whom the efficacy of PBM during wakefulness is limited.

**Graphical Abstract:**

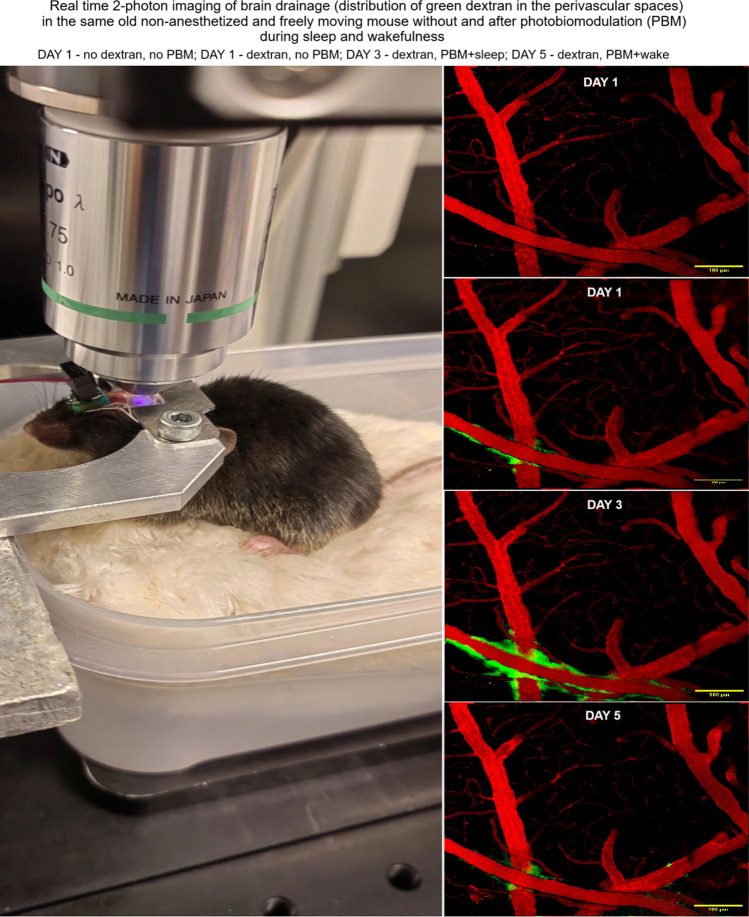

## Introduction

Age is a biological process triggering irreversible changes in the brain associated with physical and cognitive decline as well as with an increased risk of the development of neurodegenerative diseases, such as Alzheimer’s disease (AD) and Parkinson disease (PD) [1–3]. The World Health Organization estimates that the number of people aged 60 and over was 1 billion in 2019 and will increase to 1.4 billion by 2030 and 2.1 billion by 2050 [4]. Furthermore, people older 80 years are expected to triple from 2020 to 2050 [4]. On the one hand, the increase in life expectancy is associated with an improved quality of life and the health service. On the other hand, an increased longevity is associated with age-associated brain disorders, among which the most common is cognitive deficit, anxiety and depression [1, 5].

Non-pharmacological therapies have received increasing attention from researchers as a strategy to improve cognitive impairment in old patients due to the difficulties of using pharmacological therapy in this category of patients [6]. The growing number of clinical studies demonstrates that significantly functional impaired older patients have not been adequately taken into consideration in most clinical trials investigating drug therapy of neurocognitive diseases [7]. Despite the fact that in 1993 the International Council for Harmonisation of Technical Requirements for Pharmaceuticals for Human Use recommended to include frail older patients in the randomized controlled trials, these patients are still seldom included in clinical investigations [8–11]. Although old patients are overrepresented in clinicals, they are underrepresented in the clinical trials that provide the data on which clinical treatments are based [7, 12]. Due to the deficiency of clinical data, old age is often ignored during the formulation of medical guidelines [13]. It is well-known that pharmacological therapy has different effects in these vulnerable old patients: systemic diseases, metabolic and cardiovascular changes may result in different pharmacologic responses with an adverse event and risk–benefit calculus [12, 14, 15]. The weak sensitivity to pharmacological therapy in elderly patients may be associated with age-altered pharmacokinetics leading to reducing drug efficacy. Age-related changes in the liver slow down the elimination of the drug from the body, increasing the risk of toxicity [16]. Therefore, dose adjustment is crucial for elderly patients. There are a number of restrictions for medications in elderly patients, including special recommendations from the American Society for Geriatric Practice have been developed due to age-related changes in receptors and concomitant diseases [17]. For example, old patients experience the strongest side effects of complex treatment for multiple sclerosis [18]. Elderly PD patients also have a higher risk of side effects caused by levodopa [19], which underscores the need for age-appropriate therapy, in particular non-invasive methods. The benefits of dopaminergic therapy in PD diminish with age due to altered drug metabolism [20]. Nagayama et al. recently considered the effect of age on pharmacokinetics and pharmacodynamics of levodopa, and used 75 years and older to define “late elderly” patients with a specific interest [21].

Among non-pharmacological interventions, transcranial photobiomodulation (PBM) is considered as the most promising method for treating neurodegenerative diseases [22–26]. It should be noted that the overwhelming majority of studies on the therapeutic effects of PBM in improving memory, cognitive functions, energy and neuronal metabolism have been conducted on young or middle-aged animals or humans. However, there is evidence that the effectiveness of PBM in older subjects is limited [27]. Indeed, Blivet et al. did not find the significant therapeutic effects of PBM on cognitive function in AD patients over 73 [28]. Herkers et al. demonstrate no significant results in PBM-mediated reducing the motor signs of PD in old people with an average age of 72 [29]. Likewise, Bullock-Saxton et al. show that PBM did not affect cognitive function in PD patients aged 73 and over [30]. A similar trend is also observed in older people without signs of neurodegenerative diseases, in whom PBM has no effect on improving visual memory [31]. Animal studies show a similar pattern. So, Cardoso et al. did not reveal the PBM therapeutic effects on associative memory, anxiety, and locomotor activity in aged rats (20-month-old) [32]. Hosseini et al. using a model (the injection of D-galactose) of aging brain demonstrate that PBM did not improve associative memory and social interactions in affected mice [33]. Sipion et al. using 5xFAD mice (8 months at the time of testing), found no significant differences between the PBM and no PBM groups in the behavioral tests, including the Morris water maze, novel object recognition, and Y-maze [34]. They also show the no PBM effects on amyloid-beta (Aβ 1–42) load, neuronal loss or microglial response in AD mice [34]. Buendía et al. using transgenic AD mice (APP/PS1), did not observe significant effect (only a tendency) on improvement of cognitive abilities (assessed by the novel recognition test) in a murine model of AD [35]. Lutfy et al. clearly show that PBM did not ameliorate short-term special memory in 16-month-old rats [36].

Brain drainage is an important for maintaining brain homeostasis. The meningeal lymphatic vessels (MLVs) perform the function of removal of metabolites and toxins from the brain with the flow of brain fluids [37–39]. Age-related decline in the MLV functions is accompanied by the accumulation of toxic metabolites in the brain, such as Aβ associated with cognitive deficit [37–39].

MLVs are targets for PBM, which has stimulating effects on brain drainage and the removal of toxins from its tissues [38–43]. Recent studies have found that old age is a limiting factor for the effectiveness of PBM in stimulation of the MLV functions [27].

Sleep is a natural time for activation of brain drainage [44, 45]. Recent discoveries have established that the therapeutic effects of PBM during sleep are significantly higher than during wakefulness, which is associated with a stronger photo-effect on MLVs in the sleeping brain [41, 42, 46–48].

Given the accumulating evidence supporting the beneficial effects of PBM during sleep, in this study, we tested the hypothesis that aged mice can be more sensitive to the PBM effects on brain drainage during sleep vs. wakefulness. To test this hypothesis, we studied the effects of PBM during sleep and wakefulness on the content of Aβ in the brain and the meninges, as well as on cognitive function in old mice (24 months, which corresponds to over 70 years in humans [49, 50]. To study the role of sleep in brain drainage and the PBM effects on these processes in mice pf different ages, we studied the distribution of fluorescent dye in brain tissues and its lymphatic excretion, as well as the level of metabolites (Aβ 1–42, tau protein (Tau), glutamate, lactate, glucose) in the brain before and after sleep deprivation (acute and chronic) of 3-12- and 24-month-old mice treated and not treated by PBM. Additionally, we studied the mechanism of stronger PBM effects on drainage of the sleeping brain using the protocols for the in vivo and ex vivo studies of perivascular and lymphatic drainage.

## Methods

### Subjects

Male C57BL/6 mice (3-12-24-month-old, 25-28-35 g, respectively) were used in all experiments and were obtained from the National Laboratory Animal Resource Centre in Pushchino (Moscow area, Russia). The choice of mouse age is related to the evaluation of PBM efficacy in young (3 months old), middle-aged (12 months old) and in old (24 months old) mice, corresponding to 25, 42.5 and 69 years of age in humans [49, 50]. The animals were housed under standard laboratory conditions with access to food and water ad libitum. All experimental procedures were performed in accordance with the “Guide for the Care and Use of Laboratory Animals,” Directive 2010/63/EU on the Protection of Animals Used for Scientific Purposes, and the guidelines from the Ministry of Science and High Education of the Russian Federation (Nº742 from 13.11.1984), which have been approved by the Bioethics Commission of the Saratov State University (Protocol No. 12, 23.12.2023 with additions No. 14, 14.07.2025).

All animals were maintained under specific pathogen-free conditions under controlled temperature (18–22 °C) and humidity (50%–60%) and a light (08:00–20:00) and dark cycle (20:00–08:00), with access to regular rodent’s chow and sterilized tap water ad libitum.

The experiments were performed in the following groups: (1–3)—the control, including intact 3-12-24 month-old mice without acute sleep deprivation (ASD) or chronic sleep deprivation (CSD) as well without PBM; (4–6) the sham-light control groups were additionally conducted to exclude the effects of head-mounted LED platform and EEE electrodes on the results and included 3-12-24 month-old mice with LED head plate and EEG electrodes without PBM and sleep deprivation; (7–9) ASD 3-12-24-month-old mice, i.e., after ASD; (10–12) CSD 3–12-24-month-old mice, i.e., after CSD; (13–15) CSD 3-12-24-month-old mice received in parallel the 10 day course of PBM + wake; (16–18) CSD 3-12-24-month-old mice received in parallel the 10 day course of PBM + sleep; *n* = 8 in each group in all sessions of the experiments; (19) non-anesthetized and freely moving 24-month-old mice, which were adapted and included in the real time monitoring of the PBM effects during sleep and wakefulness on brain drainage using 2-photon imaging on 1, 3 and 5 days of observation on the same mouse (*n* = 5); (20–22) 24-month-old mice, which were included in ex vivo confocal analysis of brain drainage without and after PBM + wake and PBM + sleep, *n* = 5 in each group.

### Immunoassay for the detection of metabolites in brain lysates

ELISA was used to measure Aβ 1–42 (Cloud Clone, NºCEA946Mu) and Tau (Cloud Clone, NºHEB983Mu) in brain lysates from mice of different ages. Brain tissues were collected, homogenized and lysed to extract proteins, and samples were prepared according to the Cloud Clone protocol for ELISA sample preparation. Brain lysates were prepared in lysis buffer (1.5 mM KH_2_PO_4_, 8 mM Na_2_HPO_4_, 3 mM KCl, 137 mM NaCl, 0.1% Tween 20, 10 mM EDTA) at pH 7.2 with a freshly prepared protease inhibitor cocktail (Roche Applied Science).

Tissues were washed in ice-cold phosphate-buffered saline to thoroughly remove excess blood, weighed before homogenization, minced into small pieces, and homogenized in fresh lysis buffer using a glass homogenizer on ice. The resulting suspension was sonicated using a cell disruptor until the solution clarified. Homogenates were then centrifuged for 5 min at 10,000 × g. The supernatant was collected and immediately analyzed. Optical density measurements of the samples were performed at a wavelength of 450 nm (A450) using an automated ELISA microplate spectrophotometer, Epoch BioTek Instruments (BioTek Instruments, Winooski, USA).

The high-performance liquid chromatography (HPLC) was performed on an Agilent Technologies 1220 Infinity II HPLC chromatograph (Agilent Technologies, Santa Clara, USA) with a ZORBAX Eclipse C18 4.6 × 150 mm 5-Micron column and a UV detector at 254 nm for the quantitative analysis of glutamate content in brain lysates. Chromatography was conducted using a solvent system: Phase A: 20 mM phosphate buffer (pH 6.8) with 5% acetonitrile; Phase B: 80% acetonitrile in water, linear gradient 0%–50% B over 25 min. Injection volume was 10 µL, derivatizing agent was the ortho-phthalaldehyde (OPA) with mercaptoethanol or 6-aminoquinolyl-N-hydroxysuccinimidyl carbamate (AQC). Standards: Glutamate solution (L-glutamic acid, Sigma-Aldrich). Solvents: Acetonitrile, methanol, water (HPLC-grade). Sample Preparation: Centrifuge, filters (0.22 µm), autosampler vials. Derivatization: To 10 µL of cerebrospinal fluid (CSF), 20 µL of OPA solution (10 mg/mL in borate buffer, pH 9.5, with 2-mercaptoethanol) was added. The mixture was incubated for 1–2 min at room temperature in the dark. The reaction mixture was diluted to 100 µL with the mobile phase and injected into the chromatograph. Mobile Phase: Phase A: 20 mM phosphate buffer (pH 6.8) with 5% acetonitrile; Phase B: 80% acetonitrile in water. Gradient elution: 0–5 min—5% B, 5–20 min—linear increase to 50% B, 20–25 min—50% B, then return to 5% B. Flow rate: 1.0 mL/min. Column temperature: 30–40 °C. Injection volume: 10 µL. Analysis time: ~ 25–30 min per sample. UV detector at 254 nm. A calibration curve was constructed using standard glutamate solutions (0.1–100 µM). Detection limit: ~ 0.01–0.1 µM. Glutamate concentration was calculated based on peak area normalized to an internal standard using Agilent Technologies software.

The measure of the brain lactate level was performed using a spectrocolorimetric method according to the standard Abcam protocol with the L-Lactate Colorimetric/Fluorometric Assay Kit (ab65330, Abcam, UK). The reaction mixture contained the 46 µL Lactate Assay Buffer, the 2 µL Lactate Probe, the 2 µL Lactate Enzyme Mix, and the 100 µL of tested sample. The reaction mixture was incubated for 30 min at room temperature, followed by colorimetric measurement at a wavelength of 570 nm using a Cary Eclipse spectrofluorometer. Lactate concentration in the samples was determined using a calibration curve constructed from six points with lactate concentrations ranging from 0 to 10 pmol/µL. For the calibration curve, 0, 6, 12, 18, 24, and 30 µL of a standard lactate solution (100 nmol/µL) were added to the reaction mixture, incubated for 30 min at room temperature, and measured colorimetrically at 570 nm using a Cary Eclipse fluorescent spectrophotometer (Agilent, Santa Clara, California, USA).

The glucose brain level was measured according to the standard AGAT protocol (Biocont, Russia) using the Glucose-AGAT kit (Biocont, Russia, 400 determinations × 1 mL, Art. 10,757) for the quantitative determination of glucose content in brain lysates via the glucose oxidase method. Glucose oxidase oxidizes D-glucose to gluconic acid, producing hydrogen peroxide, which, under the action of peroxidase, reacts with 4-aminoantipyrine and phenol to form a red-colored compound. The intensity of the color is proportional to the glucose concentration in the sample and is measured photometrically at a wavelength of 510 nm (470–540 nm). The concentration was calculated using a calibration curve with standard glucose solutions (0.2–22.2 mM/L). Measurements were performed on a spectrophotometer at 510 nm using a Cary Eclipse fluorescent spectrophotometer (Agilent, Santa Clara, California, USA).

The level of the studied metabolites in the brain was studied in 3-12-24-month old mice before and after ASD or CSD, as well as before and after the 10-day course of PBM in CSD mice of different ages.

### Ex vivo optical analysis of brain drainage

An amount of 5 μL 0.5% solution in physiologic 0.9% saline of the Fluorescein Isothiocyanate-Dextran 70 kDa (FITCD, Sigma-Aldrich, St Luis, USA) was injected into the right lateral ventricle (AP—1.0 mm; ML—1.4 mm; DV—3.5 mm) at a rate of 0.1 μL/min using microinjector (Stoelting, St. Luis, USA) with a Hamilton syringe with a 29-G needle (Hamilton Bonaduz AG, Bonaduz, Switzerland) via a polyethylene catheter. The implantation of catheter (PE-10, 0.28 mm ID × 0.61 mm OD, Scientific Commodities Inc., Lake Havasu City, Arizona, USA) into the right lateral ventricle was preformed according to the protocol reported by Devos and Miller [51].

The optical imaging of the FITCD distribution in the dorsal and ventral parts of the brain as well as tracer accumulation in the deep cervical lymph nodes (dcLNs) was performed 1 h after dye administration using a homemade optical instrument based on the monochrome camera acA2040–2090 μm (Basler, Ahrensburg, Germany) and a 50 mm 2.8 C-mount CCTV objective lens (Tamron, Japan). The lens was attached to the camera with a 15 mm extension tube to ensure macro imaging with a 23.3 to 31.8 mm field of view depending of the lens focusing ring adjustment. The lens was mounted on the vertical manual translation stage (Standa, Lithuania) above a Petri dish, where samples were submerged in a buffer solution. The top surface of each sample was covered with a 25 mm × 50 mm × 0.17 mm cover glass. The slider with filter sets (49019, 49002, Chroma Technology, USA) was placed just below the objective lens. Each filter set was illuminated with homemade condensers with 1 W LEDs (635 nm for 49019 and 460 nm for 49002) to ensure uniform illumination over the camera field of view. Led illuminators were synchronized with the camera “fire” output.

The camera resolution was 2048 × 2048 pixels at 12 bit grayscale. Images were acquired in a dark room at a constant exposure time of 200 ms, and other settings were kept unchanged for all samples. Image acquisition and Instruments, Eagan, United States) and the Fiji open-source image processing package [52]. Image processing procedures were identical for each pair of images (control and laser-treated samples) for each channel to ensure an accurate comparison of the fluorescence intensity.

Brain drainage was analyzed before and after ASD or CSD in 3-12-24-month old mice.

Additionally, using the same protocol of intraventricular administration of FITCD via a catheter, the PBM effects on brain drainage were studied by ex vivo confocal (LEICA TCS SP8, Leica Microsystems, Wetzlar, Germany) analysis of FITCD accumulation in dcLNs 1 h after its administration. The cerebral vessels were filled by Evans Blue dye injected into the tail vein (Sigma Chemical Co., St. Louis, Missouri, 2 mg/bodyweight, 1% solution in physiologic 0.9% saline) immediately after administration of FITCD. After imaging, the fluorescence intensity of FITCD in dcLNs (a.u.) was measured using FIJI software.

### In vivo optical analysis of brain drainage

Here we used the modified previously published our methods [53]. In the first step (the training period), 24-month-old mice were placed daily in an experimental setting, including a large cage (300 mm × 180 mm × 150 mm) with a small individual box (150 mm × 100 mm × 50 mm) for sleep. A large cage allows mouse to create a more comfortable environment that to feel the safe. The small box was not fixed and can be moved in any direction for maximum comfort and to choose the optimal place to sleep. It is also important for effective training of head-fixed mouse to sleep under microscope, because animals itself can adapt location of small box for optimal body position for sleep. The mattress of small individual box was composed of bedding from home cage to retain the mouse scent, cotton, polyurethane foam, wrapped in soft and tender material, which was chosen because of its resemblance to the hair of mice. The length of the training period (from one month to two) was selected individually and depended on the possibility of the mouse falling asleep under a 2-photon microscope without a head-fixation platform. Mice that did not adapt to the experimental conditions were excluded from the studies.

In the second step (the preparation period), we selected only mice, which could demonstrate NREM and REM sleep under a 2-photon microscope during training period. Ten days before experiments, surgical procedures were performed, including (i) implantation of chronic polyethylene catheters into the right lateral ventricle (AP, 1.0 mm; ML,− 1.4 mm; DV, 3.5 mm) for the injection of FITCD (5 μL, at a rate of 0.1 μL/ min, 0.5% solution in saline, Sigma-Aldrich, USA); (ii) implantation of the EEG electrodes, LED head plate; (iii) preparation of the chronic cranial window with a diameter of 3 mm using this method [54].

Optical monitoring of brain drainage in non-anesthetized (since anesthesia alters brain drainage [55]) 24-month-old mice was performed 30 min after intraventricular injection of FITCD. Freely moving mice were fixed using the head-plate with a fixing system and placed under multiphoton microscope (Nikon A1R MP, Nikon Instruments Inc., Tokyo, Japan). Images were acquired using a Spectraphysics Insight X3 pulsed laser, excitation wavelength 970 nm). Images of the chronic window were recorded using a 20 × 0.75 objective over 60 min with 10 min interval; each image was composed of 1 field of view (525 µm × 525 µm) of 1024 × 1024 points. The brain image was acquired as a stack of 25 layers registered in 2.5 µm depth increments, and then presented as a maximum intensity projection of all layers. The multiphoton images were captured in two channels: 488 nm excitation/525 emission was used to image FITCD distribution.

### Modeling sleep deprivation

**Fig. 1:**
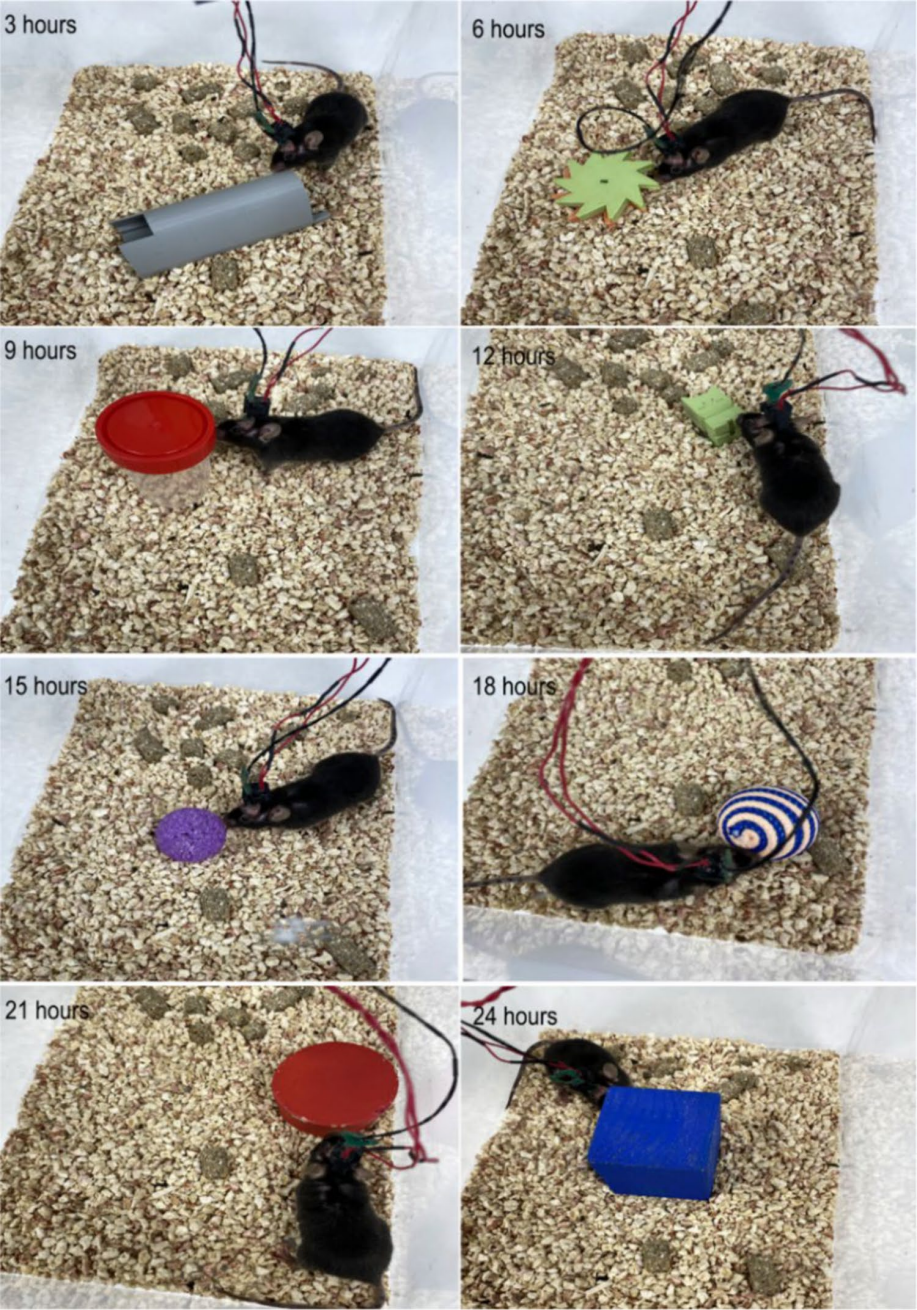
Modeling sleep deprivation by presenting a new object in the animal cage

Sleep deprivation in mice was performed according to the method described in these publications [56, 57]. The choice of this method is due to the fact that it is recognized as non-stressful, which is not accompanied by an increase in the level of corticosterone in the blood [56]. We used the method of ASD for one day by presenting new objects in the animal’s cage, as well as CSD for 10 days, when sleep was excluded for 3 h from 5 to 8 pm using the same method by adding new objects to the cage (Fig. 1). The time for deprivation was chosen based on observations of the behavior of mice, when they prefer to sleep during the light phase (08:00–20:00) of the vivarium cycle.

**Fig. 2:**
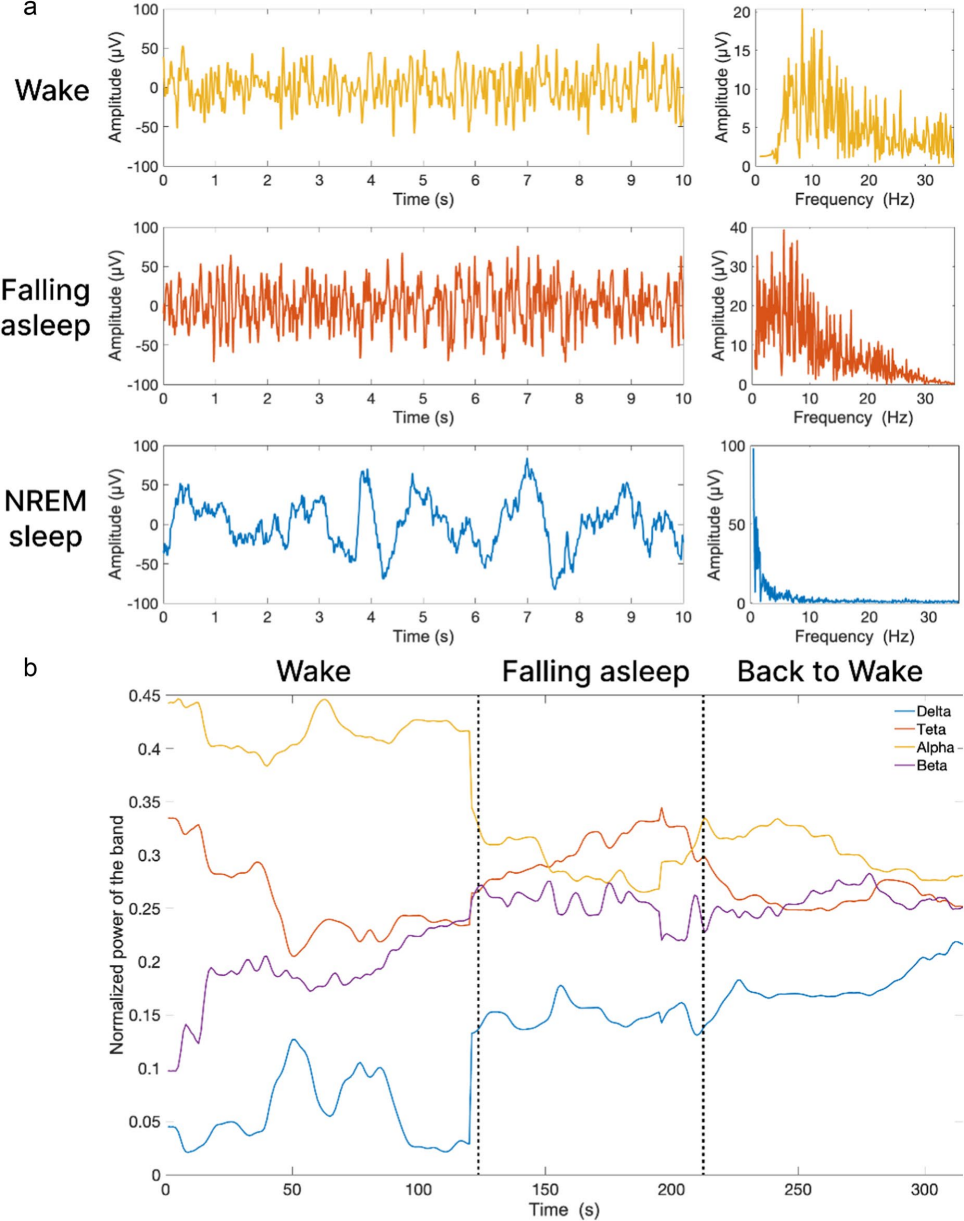
EEG validation of sleep deprivation. **a** EEG patterns of different sleep stages: wake (yellow), falling asleep (orange), and NREM sleep (blue). The wake state is characterized by a dominant alpha rhythm (8–12.5 Hz); the falling asleep state is marked by an increase in theta rhythm (4–7.5 Hz); and NREM sleep is defined by a dominant delta rhythm (0.5–3.5 Hz); **b** Spectral band power as a function of time. The transition from wake to falling asleep is defined by a decrease in alpha band power (yellow) and an increase in theta band power (orange). The reverse transition (from “falling asleep” to “back to wake”) is characterized by a decrease in theta band power and an increase in alpha band power

Figure 2 shows EEG validation of sleep deprivation. The EEG transitions from wakefulness (alpha rhythm) to drowsiness (theta rhythm) were determined when the mice wanted to sleep and when a new object was presented they woke up (the alpha rhythm appeared again).

### Serum corticosterone assay

To exclude severe stress during sleep deprivation, the level of corticosterone in the blood was additionally assessed in 3-12-24-month-old-mice. Upon completion of ASD, CSD, or at normal condition, mice (*n* = 8 in each group) was anesthetized with 1% isoflurane (Sigma-Aldrich, St Luis, USA, at rate 1 L/min N_2_O/O_2_—70/30 ratio). The blood was collected from tail nicks into Microvettes (Sarstedt, Germany) at 08:00 am (circadian rhythm nadir) [58]. The method of tail snip blood sampling causes minimal changes in the corticosterone levels what determined our choice of this method of blood collection [59]. Afterward, the blood was coagulated, centrifuged and the supernatant was collected. A commercial ELISA kit (ADI-900-097, Enzo Life Sciences, Inc., Farmingdale, NY, USA) was used according to manufacturer’s specifications to measure the serum corticosterone levels (in duplicate), using an automated ELISA microplate spectrophotometer, Epoch BioTek Instruments (BioTek Instruments, Winooski, USA).

### PBM during wakefulness and sleep

A two-channel cortical EEG/one-channel electromyogram (Pinnacle Technology, Taipei, China) was recorded. The mice were implanted with two silver electrodes (tip diameter: 2–3 µm) located at a depth of 150 µm in coordinates (L: 2.0 mm and P: 2 mm) from the bregma on either side of the midline under inhalation anesthesia with 1% isoflurane (Sigma-Aldrich, St Luis, USA, at rate 1 L/min N_2_O/O_2_—70/30 ratio). An EMG lead was inserted in the neck muscle. The mice were allowed 10 days to recover from surgery prior to beginning the experiment.

The PBM was performed with 3835 SMD LED (central wavelength 1050 nm and spectrum width of 50 nm) and with output power of 50 mW. The LED driver was controlled with pulse width modulation (PWM) output of the EEG instrument microcontroller. The LED was connected to the instrument with 0.3 m long 2 wire flexible cable and mounted into miniature 3D printed frame with a pair of cylindrical magnets 3 mm in diameter and of 3 mm height each. That allows for easy attachment of the LED frame to M3 steel washer glued at mouse skull surface. LED operates in PWM mode at 1 kHz modulation frequency. The washer hole of 3.5 mm in diameter acts as an aperture limiting PBM area to 0.1 cm^2^ at the skull surface.

LED output power of optical radiation is 50 mW and the area limited with a washer is 0.1 cm^2^ that corresponds to 500 mW/cm^2^ of power density at continuous wave operation. To minimize thermal effect optical radiation PBM was performed in pulsed regime with PWM duty of 2% that corresponds to 10 mW/cm^2^ or 1% of maximal permissible exposure (MPE) for skin following ANSI-Z136 standard. For 17 min (1020 s) long procedure at 2% PWM duty the LED delivers 10 J/cm^2^ dose over irradiated skull surface. The instrument was controlled with a PC with software developed using LabVIEW (National instruments, Texas, USA). The software enables EEG recording, monitoring of the instrument operation and remote configuration of non-rapid eyes movement (NREM) sleep detection and PBM procedure. The EEG signal was digitized at 2.4 kSa/s, and filtered with a fifth-order digital Butterworth bandpass filter with the lower cutoff frequency of 0.5 Hz and the upper one of 250 Hz. For each 1 s long record Fast Fourier transformation was used to estimate power of EEG signal within frequency bands delta (0.5–3.5 Hz), theta (4–7.5 Hz), alpha (8–12.5 Hz) and beta (13–35 Hz), denoted as PΔ, PΘ, PA and PB, respectively together with the integral spectral power PΣ of the signal within the band 0.5–35 Hz. 15 min after EEG and LED were connected to the mouse, the peak value of PΣ in wake conditions was measured and stored as a threshold value for sleep detector Pw. The 1 s record is scored as NREM sleep if the logic value of NREM = (PΔ > PA) & (PΔ > PB) & (PΘ > PA) & (PΘ > PB) & (PΔ > (PA + PB) & (PΔ > PΘ) & (Pw < PΣ) is “true.” When 20% of 30 s epoch (6 of 30 consequent 1 s records) were scored as NREM [57] then NREM sleep stage of animal was detected, and PBM LED is automatically turned on to run for 17 min at given PBM. Once configured the instrument was operated autonomously, logging its operations to be monitored via Wi-Fi connection. Wakefulness, NREM and rapid eye movement (REM) sleep were defined as described in our previous studies, where we demonstrate the EEG patterns and spectrum characteristics of wakefulness, NREM and REM sleep in mice [38, 41, 42, 60, 61]. Sleep scoring in mice has been extensively discussed in literature. Representative spectra of mice EEG for NREM, REM and wake states can be found in [62–64]. Slow wave activity within delta frequency band 0.5–3.5 Hz is the key for NREM sleep scoring.

Measurement of the PBM-mediated thermal impact, a type A-K3 thermocouple (Ellab, Hillerød, Denmark) was used to measure the skull temperature. The thermocouple was placed subcutaneously 2 mm lateral to the bregma in the irradiated zone. A burr hole was drilled under inhalation anesthesia (1% isoflurane at 1 L/min N_2_O/O_2_—70:30). To measure the brain surface temperature under the 1050 nm LED irradiation, the medial part of the left temporal muscle was detached from the skull bone, a small burr hole was drilled into the temporal bone, and a flexible thermocouple probe (IT-23, 0.23 mm diam, Physitemp Instruments LLC, NJ, USA) was introduced between the parietal bone and brain into the epidural space. Brain surface temperature was measured before and during PBM in 5 min increments using a hand-held thermometer (BAT-7001H, Physitemp Instruments LLC, NJ, USA).

The results showed that the temperature on the surface of the skull increased slightly (36.00 ± 0.12 vs. 35.94 ± 0.15, not statistically significant (ns), *n* = 5, the ANOVA test with the post hoc Duncan test). However, the temperature on the surface of the brain did not change after PBM (37.11 ± 0.10 vs. 37.12 ± 0.12, ns, the ANOVA test with the post hoc Duncan test, *n* = 5), which is consistent with our earlier results [40].

To assess the transmission of light through the skull, the spectra of total (Tt) transmission of the skull samples taken at the region there PBM was performed, were measured for 3-12-24-month-old mice (*n* = 5 in each group) in the wavelength range from 900 to 1500 nm using a spectrophotometer UV-3600 with an integrating sphere LISR-3100 (Shimadzu, Japan). A BaSO_4_-based diffuse reflection standard was used to calibrate the spectrophotometer. The resulting transmission at 1050 nm was estimated as 60 ± 5%, 47 ± 5%, 39 ± 5% for 3–12-24 month old mice, respectively.

The washer together with LED frame (weight 0.6 g, which is only 2.4%, 2.2% and 1.6% of the body weight of 3-12-24 month old mice, respectively) was fixed in the region of the parietal and interparietal bones using dental acrylic (Zhermack SpA, Badia Polesine, Italia) under inhalation anesthesia with 1% isoflurane (Sigma-Aldrich, St Luis, USA, at rate 1 L/min N_2_O/O_2_—70/30 ratio). The LED was fixed to the head plate with two screws. It should be noted that the low weight of the LED platform, the adaptation of mice to the experiment, the absence of changes in the plasma corticosterone level, and the choice of the non-stressful method of CSD (3 h per day over 10 days) indicate that stress had no effect on brain drainage and the removal of toxins from its tissues.

The PBM performed: (1) daily during 10 days under EEG monitoring of wakefulness or NREM in 24-month-old mice for the study of the PBM effects on cognitive function and clearance of Aβ (Sect. 3.1); (2) daily during 10 days under EEG monitoring of wakefulness or NREM daily during 10 days under EEG monitoring of wakefulness or NREM in CSD 3-12-24-month old mice for the study of the PBM effects on clearance of brain metabolites (Sect. 3.2.2); (3) PBM performed (single application) under EEG monitoring of wakefulness or NREM on 3rd and 5th days of real-time 2-photon imaging of brain drainage in the same 24-month-old mouse (Sect. 5); (4) PBM performed (single application) under EEG monitoring of wakefulness or NREM for the ex vivo confocal analysis of the PBM effects on brain drainage (Sect. 5).

### Immunohistochemistry and confocal imaging

The Aβ 1–42 levels in the whole brain and in its meninges as well as the content of FITCD in dcLNs were measured using the standard abcam protocols for free-floating sections and a confocal imaging (LEICA TCS SP8, Leica Microsystems, Wetzlar, Germany). The whole brains, the meninges and dcLNs were collected. Evans blue dye (1%, Sigma-Aldrich, St Louis, MI, USA) was injected intravenously to fill the cerebral vessels. The brains and the meninges were fixed for 48 h in a 4% saline solution-buffered formalin; the dcLNs were fixed in a 4% saline solution-buffered formalin overnight at 4 °C, and then fixed in 2% agarose; the brains and the dcLNs were cut on the slices with a thickness of 40–50 microns using a vibrotome Leica VT1000 S (Leica Microsystems, Wetzlar, Germany). The nonspecific activity was blocked by 2 h incubation at room temperature with 10% bovine serum albumin in a solution of 0.2% Triton X-100 in PBS. Solubilization of cell membranes was carried out during 1 h incubation at room temperature in a solution of 1% Triton X-100 in PBS. Incubation with primary antibodies in a 1:500 dilution took place overnight at 4 °C with mouse anti-Aβ 1–42 peptide antibody (1:200; no. MAA946Ge21, Cloud Clone, Wuhan, China), rabbit anti- NG2 antibody (1:500; ab217672, Abcam, Waltham, USA) when studying sections of the brain and its meninges.

At all stages, the samples were washed 3–4 times for 5 min incubation in a washing solution. After that, the corresponding secondary antibodies goat anti-mouse IgG (H + L) Alexa Flour 488, goat anti-mouse IgG (H + L) Alexa Flour 555, goat anti-rabbit IgG (H + L) Alexa Flour 555 (Invitrogen, Molecular Probes, Eugene, Oregon, USA) were applied. At the final stage, the sections were transferred to the glass and 15 µL of mounting liquid (50% glycerin in PBS) was applied to the section. The slices were covered with a cover glass and confocal microscopy was performed.

The sections of the brains, the meninges and dcLNs were visualized using a confocal microscope LEICA TCS SP8 (Leica Microsystems, Wetzlar, Germany) with a × 20 lens (0.75 NA) or a × 100 lens for immersion in oil (0.45 NA). DAPI, Alexa Fluor 488 and Alexa Fluor 555 were excited with excitation wavelengths of 405 nm, 488 nm, and 561 nm, respectively. Evans Blue were excited with the same excitation wavelength of 647 nm. Three-dimensional visualization data were collected by software 3.0.16120.2 LAS X (Leica Microsystems, Germany) and analyzed using Fiji software 2.0.0 (Open-source image processing software).

### Behavioral testing

To assess cognitive function in mice of different ages, we used the modified method of the Pavlovian conditioning using the combination “light-food” and an automated operating wall published in Refs. [42, 65]. A reward (12 mg seeds of sunflowers, Grums, Minsk, Belarus) was given if the mouse placed its head into the hall (the number of head entries per session during 20 min was counted), above which a green light was switched on. Mice were placed in an operant chamber every day and a camera was used to record the formation of a conditioned reflex which was estimated as the receipt of a reward within 15 s after the mouse was placed in the operative wall. This test assessed the number of sessions (days) required to develop the stable conditioned reflex “light-food” and the number of head entries in the hall with receipt of reward.

The choice of the method of the Pavlovian conditioning for assessing cognitive function is based on our extensive previous experience [66]. Conditioned reflexes are formed by activating a large number of nerve centers, including motivation (hunger and interest), memory (especially, the formation of memory in the hippocampus playing a crucial role for forming, consolidating, and retrieving memory as well as in transferring information from short-term to long-term memory during training process), and emotions (positive reinforcement in the form of food reward) [67, 68]. The transition short-memory to long-term memory known as memory consolidation that is a biological process where transient neural activity patterns become more stable and enduring, forming lasting memories. In Pavlov’s classical conditioning, a lever (conditioned stimulus, CS) paired with food (unconditioned stimulus, US) initially elicits a short-term memory of that association [69, 70]. Repeated pairings of CS and US strengthen the connections between neurons involved in the memory trace. This involves changes in the structure and function of synapses, the connections between neurons. The hippocampus plays a crucial role in the initial stages of memory formation, including the formation of conditioned reflexes. It helps retrieve information from working memory and begin the process of establishing new neural connections. The formation of long-term memories is not instantaneous. It involves a time-dependent process where detailed memories can be transformed into more generalized or semantic representations over time [69, 70]. The emotionally significant events are an important factor in the formation of conditioned reflexes, where a neutral stimulus becomes a conditioned stimulus, eliciting a conditioned emotional response. Strong emotional responses influence the consolidation process, potentially strengthening or weakening memory depending on the context and intensity [71]. Sleep plays a critical role in memory consolidation, allowing the brain to further strengthen and reorganize neural connections [72]. The formation of conditioned reflexes is related to higher nervous activity, which allows for a full, long-term, and automatic assessment of cognitive function in a home cage, i.e., without the presence of an experimenter. Note that traditionally and frequently used tests, such as the open field, the new object recognition, Y-maze tests, are not sensitive enough to analyze age-related or early changes in cognitive function, which we have shown previously [63] and which is consistent with the results of other studies [34, 35, 73].

The operant wall was controlled by specialized software developed using Arduino IDE and LabVIEW. The microcontroller processes signals from infrared sensors, levers, and LEDs, while the graphical user interface enables the initiation and monitoring of experiments, data recording on a PC, and real-time visualization of results. The software consists of three main modules: initialization of the user interface and data logging, real-time signal processing from the operant wall, and bidirectional communication with the microcontroller.

The system supports three operational modes: a training mode, in which food is dispensed upon activation of any infrared sensor; an experimental mode with LED-based choice, where the animal must select the compartment indicated by an illuminated LED to receive a food reward; and a lever-press mode, which requires the animal to press one of the side-mounted levers to trigger food delivery. The experimental algorithm includes launching the selected mode, buffering and processing the data and displaying results on the PC screen.

### Statistical analysis, randomization and blinding

All statistical analyses and box plot visualization performed using the programming platform MATLAB R2023b, ImageJ, Microsoft Office Excel, and STATISTICA 10 for Windows software. The results were reported as a mean value ± standard error of the mean (SEM). The difference from the basal values, age differences and the influence of factors such as sleep deficit and the PBM course were assessed using ANOVA test with the post hoc Duncan and Bonferroni corrections. The significance levels were set at *p* < 0.05 for all analyses. In cases where the results of the Bonferroni and Duncan corrections were different, paired comparisons were additionally performed using the Mann–Whitney–Wilcoxon signed-rank method.

Animals from different cages, but within the same experimental group, were selected to assure randomization. Experimenters were blinded to the identity of experimental groups from the time of euthanasia until the end of data collection and analysis for at least one of the independent experiments. Mice behavior was recorded by a digital video camera mounted above the conditioning chamber and analysis of head entries was manually scored by a blinded experimenter using the automated operant wall with the specialized software developed using Arduino IDE and LabVIEW. The analysis of number of sessions in the behavior testing as well as in scoring of fluorescence was performed by a blinded experimenter. A double-blind approach was used for immunofluorescence confocal microscopy of the PBM effects on brain drainage. The blinded experimenter analyzing the fluorescence images did not know which images belonged to the PBM groups and which to the control group.

## Results

### PBM + sleep but not PBM + wake improves clearance of Aβ and cognitive function in old mice

**Fig. 3:**
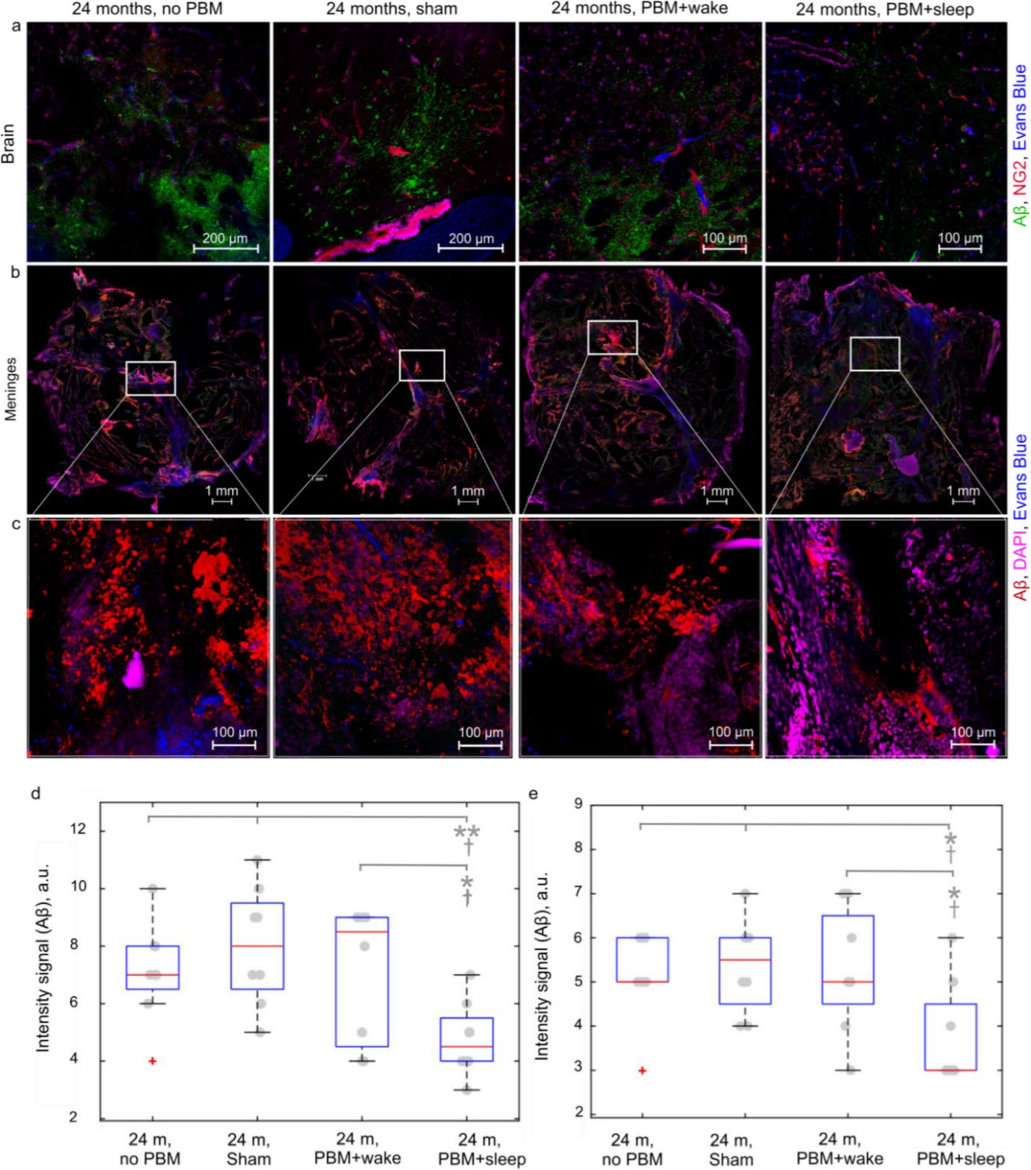
Effects of the 10-day course of PBM + sleep or PBM + wake on the content of Aβ in the brain and the meninges in 24-month-old mice. **a** Representative images of presence of Aβ (green) in the cortex of old mice from the tested groups, the blood vessels are filled with Evans Blue (blue), the pericytes are labeled with NG2 (red), DAPI (violet). **b** and **c** Representative images of presence of Aβ (red) in the whole meninges (**b**) and a region of interest (ROI) with a high magnification (**c**) in old mice from the tested groups, DAPI (violet), the blood vessels are filled with Evans Blue (blue). **d** and **e** Quantitative analysis of the intensity of the fluorescent signal from Aβ in the brain (**d**) and the meninges (**e**) in the tested groups, *p < 0.05, **p < 0.01; 8 in each group; the ANOVA test with the post hoc Duncan test; ^Ɨ^*p* < 0.05, ^ƗƗ^*p* < 0.01 for Bonferroni correction

Since our previous studies failed to show the effects of PBM + wake on the high brain Aβ levels and a cognitive dysfunction in old mice (24 months) [27], here we tested whether sleep could enhance the efficiency of PBM. Figures 3a–c clearly demonstrate that the 10-day course of PBM + sleep but not PBM + wake was associated with an improved Aβ levels in both the brain and the meninges in 24-month-old mice. Indeed, PBM + sleep reduced the Aβ level in the brain by 1.6 times (*p* < 0.01) and by 1.4 times (*p* < 0.05) in the meninges compared to animals without PBM (in the brain: 4.80 ± 0.45 a.u. vs. 7.62 ± 0.48 a.u., *p* < 0.05; in the meninges: 3.83 ± 0.41 a.u. vs. 5.44 ± 0.20 a.u., *p* < 0.05, *n* = 8 in each group, the ANOVA test with the post hoc Duncan test) (Fig. 3d and e). On the contrary, PBM + wake did not affect the Aβ levels in the brain and the meninges of old mice (in the brain: 7.12 ± 0.83 a.u. and 7.62 ± 0.48 a.u., ns; in the meninges: 5.41 ± 0.49 a.u. vs. 5.44 ± 0.20 a.u., ns, *n* = 8 in each group, the ANOVA test with the post hoc Duncan test and Bonferroni corrections) (Fig. 3d and e).

**Fig. 4:**
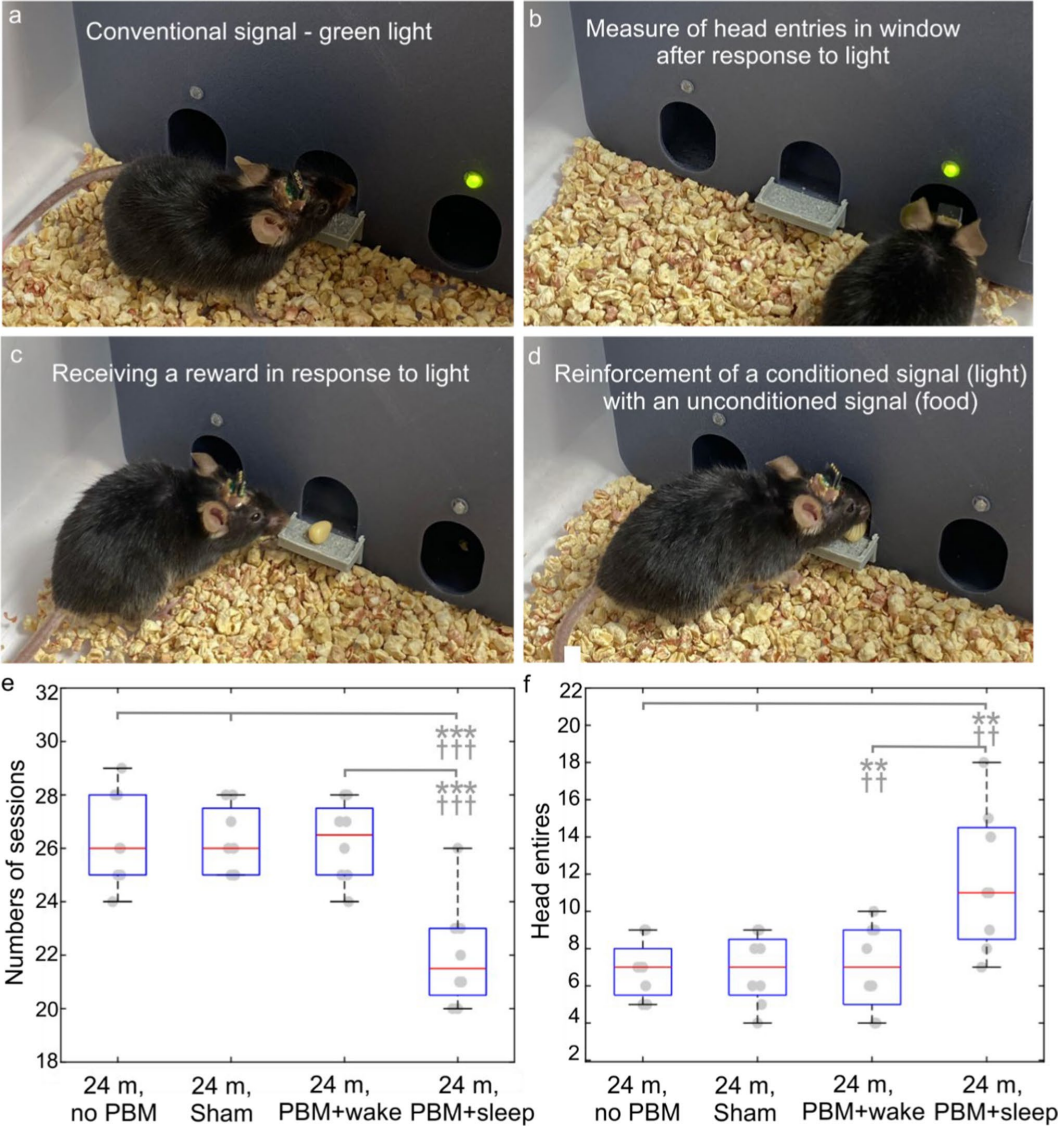
Effects of the 10-day course of PBM during sleep and wakefulness on the formation of the Pavlovian conditioning trials in 24-month-old mice. **a–d** Illustration of the operative wall used for the Pavlovian conditioning and stages of formation of the conditioned reflex “light-food.” **e** and **f** Quantitative analysis of the effects of the 10-day course of PBM + wake or PBM + sleep on formation of the conditioned reflex “light-food” in the tested groups, including evaluation of the number of sessions required to reach the conditioned reflex (**e**) and the number of head entries (**c**) in the food dispenser for rewards, *n* = 8 in each group; **p < 0.01, ***p < 0.001 for the ANOVA test with the post hoc Duncan test; ^Ɨ^*p* < 0.05, ^ƗƗ^*p* < 0.01, ^ƗƗƗ^*p* < 0.001 for Bonferroni correction

Similar results were obtained in the study of the PBM effects on an improvement of cognitive function in 24-month-old mice (Fig. 4a–d). The results of the Pavlovian conditioning trials clearly show that in the PBM + sleep group compared to the control, the number of training sessions decreased by 1.2 times (*p* < 0.001) and the number of rewards increased by 1.7 times (*p* < 0.01), indicating the positive effects of PBM + sleep on an improvement of cognitive function in old mice (the number of training sessions: 22.03 ± 0.71 vs. 26.38 ± 0.63, *p* < 0.001; the number of head entries: 11.63 ± 1.64 vs. 6.88 ± 0.55, *n* = 8 in each group, the ANOVA test with the post hoc Duncan test) (Fig. 3e and f). However, PBM + wake did not affect the learning of old mice (the number of training sessions: 26.25 ± 0.53 vs. 26.38 ± 0.63, ns; the number of head entries: 7.00 ± 0.82 vs. 6.88 ± 0.55, ns, *n* = 8 in each group, the results of testing the null hypothesis by two tests the ANOVA with the post hoc Duncan test and Bonferroni correction were the same) (Fig. 4e and f).

No changes were found between the control and sham groups in both the content of Aβ in the brain and the meninges (in the brain: 7.62 ± 0.48 a.u. in the control and 7.57 ± 0.69 a.u. in the sham group, ns; in the meninges: 5.44 ± 0.20 a.u. in the control and 5.29 ± 0.42 a.u. in the sham group, ns, *n* = 8 in each group, the ANOVA test with the post hoc Duncan test) as well as in performing a cognitive test (the number of training sessions: 26.38 ± 0.63 in the control and 26.25 ± 0.45 in the sham, ns; the number of head entries: 6.88 ± 0.55 in the control and 7.00 ± 0.67 in the sham group, ns, *n* = 8 in each group, the ANOVA test with the post hoc Duncan test).

Thus, the results of this series of experiments confirmed the hypothesis that sleep improves the stimulating effects of PBM on clearance of Aβ and cognitive function in old mice.

### Sleep deprivation suppresses brain drainage and removal of metabolites from the brain in age-related manner

#### Effect of ASD and CSD on brain drainage in mice of different ages

Brain drainage, which is activated during sleep, plays a key role in removing metabolites from brain tissues with fluid flow [38, 41, 42, 44–48]. Therefore, in the next stages of the study, we examined the effects of ASD and CSD on brain drainage and the content of brain metabolites. Furthermore, we analyzed the levels of typical brain metabolites before and after CSD + the 10-day course of PBM (wake or sleep) in mice of different ages.

**Fig. 5:**
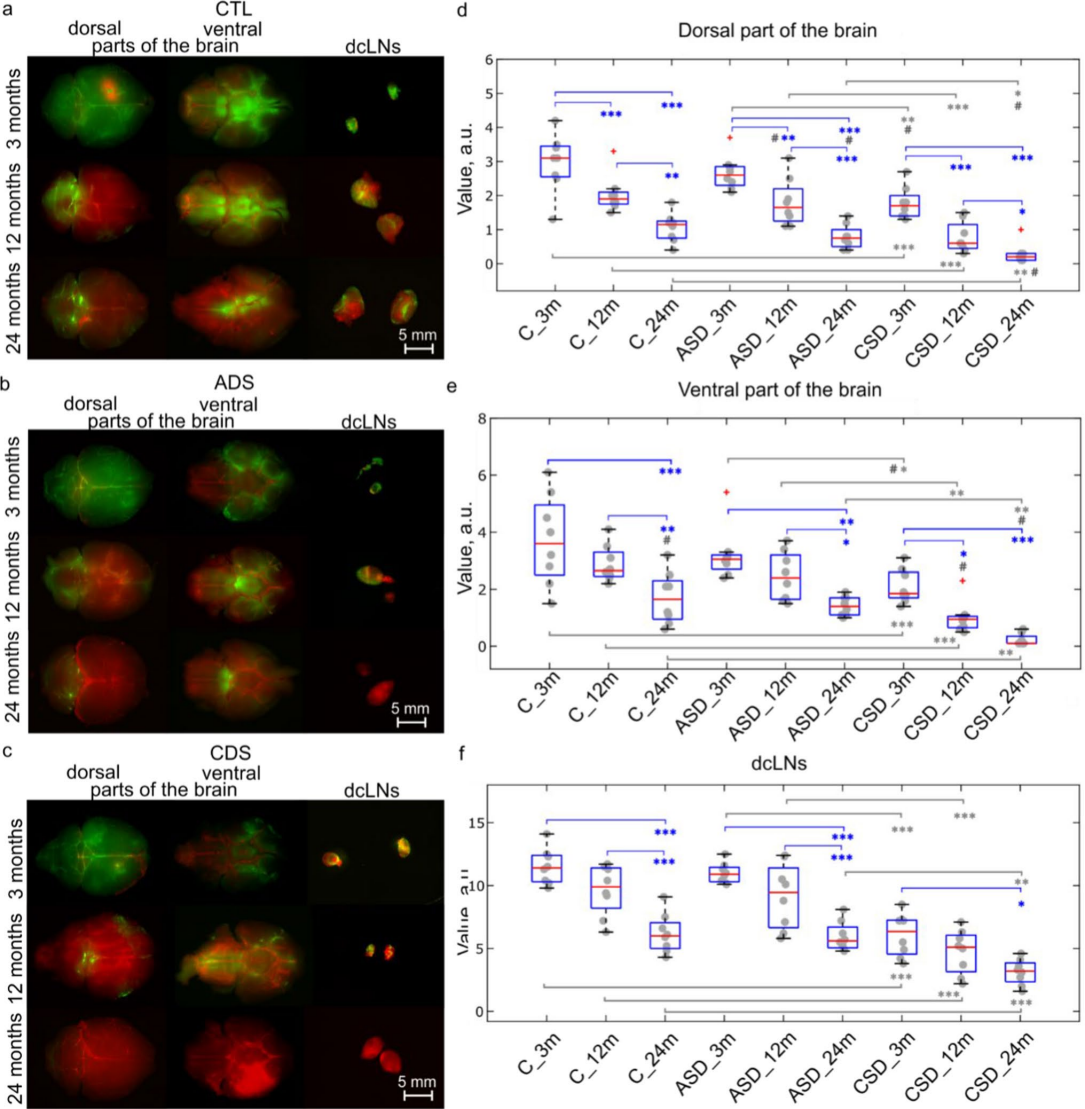
Effects of ASD and CSD on brain drainage in mice of different ages. **a–c** Representative images of FITCD (green) distribution on ventral and dorsal parts of the brain and FITCD accumulation in dcLNs in 3-, 12- and 24-month-old mice from the control, the ASD and the CSD groups. The cerebral blood vessels were filled with Evans Blue (red); **d–f** Quantitative analysis of the intensity of the fluorescent signal from FITCD in ventral part of the brain (**d**), dorsal part of the brain (**e**) and in dcLNs (**f**) in the tested groups, *p < 0.05, **p < 0.01, ***p < 0.001; *n* = 8 in each group; the ANOVA test with the post hoc Duncan test (*p < 0.05, **p < 0.01, ***p < 0.001), additional pairwise comparisons by Mann–Whitney–Wilcoxon test (^#^*p* < 0.05), *n* = 8 in each group

Figure 5a–f clearly demonstrate that brain drainage was significantly reduced in 24-month-old mice compared with 3-month-old mice (by 2.2 times, *p* < 0.001 and by 2.7 times, *p* < 0.001 in ventral and dorsal parts of the brain; by 1.8 times, *p* < 0.001 in dcLNs) and 12-month-old mice (by 1.7 times, *p* < 0.01 and by 0.6 times, *p* < 0.01 in ventral and dorsal parts of the brain, by 1.5 times, *p* < 0.001 in dcLNs). There were no differences in brain drainage between 3- and 12-month-old mice.

Quantitative analysis of age differences in basal brain drainage is presented in Fig. 5d and f (ventral part of the brain: 1.73 ± 0.32 a.u. vs. 3.72 ± 0.56 a.u., *p* < 0.001 between 24- and 3-month-old mice; 1.73 ± 0.32 a.u. vs. 2.90 ± 0.23 a.u., *p* < 0.01 between 24- and 12-month-old mice; dorsal part of the brain: 1.12 ± 0.15 a.u. vs. 3.03 ± 0.30 a.u., *p* < 0.001 between 24- and 3-month-old mice; 1.12 ± 0.15 a.u. vs. 1.90 ± 0.09 a.u., *p* < 0.01 between 24- and 12-month-old mice; dcLNs: 6.25 ± 0.55 a.u. vs. 11.51 ± 0.53 a.u., *p* < 0.001 between 24- and 3-month-old mice; 6.25 ± 0.55 a.u. vs. 9.67 ± 0.71 a.u., *p* < 0.001 between 24- and 12-month-old mice; *n* = 8 in each group; the ANOVA test with the post hoc Duncan and Bonferroni test supplemented by the Mann–Whitney–Wilcoxon signed-rank test in the case of non-Gaussian statistics or the absence of significant differences by one of the post hoc tests).

CSD but not ASD suppressed brain drainage in age-related manner (Fig. 5a–f). Indeed, after CSD, brain drainage was reduced in 24-month-old mice by 7.8 (*p* < 0.01) and by 5.6 (*p* < 0.01) in ventral and dorsal parts of the brain as well as by 2.0 times (*p* < 0.001) in dcLNs; in 12-month-old mice by 3.5 (*p* < 0.001) and by 2.2 (*p* < 0.001) in ventral and dorsal parts of the brain as well as by 2.0 times (*p* < 0.001) in dcLNs; in 3-month-old mice by 1.7 (*p* < 0.001) and by 1.7 (*p* < 0.001) in ventral and dorsal parts of the brain as well as by 1.9 times (*p* < 0.001) in dcLNs. It should be noted that ASD did not affect brain drainage in mice of different ages (Fig. 4a–f).

Figure 5d and f show quantitative analysis of changes in brain drainage after CSD in mice of different ages (24-month-old mice after and before CSD, ventral part of the brain: 0.22 ± 0.07 a.u. vs. 1.73 ± 0.32 a.u., *p* < 0.01; dorsal part of the brain: 0.20 ± 0.03 a.u. vs. 1.12 ± 0.15 a.u., *p* < 0.01; dcLNs: 3.11 ± 0.36 a.u. vs. 6.25 ± 0.55 a.u., *p* < 0.001; 12-month-old mice after and before CSD, ventral part of the brain: 0.83 ± 0.09 a.u. vs. 2.90 ± 0.23 a.u., *p* < 0.001; dorsal part of the brain: 0.88 ± 0.16 a.u. vs. 1.90 ± 0.09 a.u., *p* < 0.001; dcLNs: 4.73 ± 0.62 a.u. vs. 9.67 ± 0.71 a.u., *p* < 0.001; 3-month-old mice after and before CSD, 2.11 ± 0.21 a.u. vs. 3.72 ± 0.56 a.u., *p* < 0.01; dorsal part of the brain: 1.80 ± 0.17 a.u. vs. 3.03 ± 0.30 a.u., *p* < 0.01; dcLNs: 6.12 ± 0.60 a.u. vs. 11.51 ± 0.53 a.u., *p* < 0.001; *n* = 8 in each group; the ANOVA test with the post hoc Duncan, Bonferroni test and the Mann–Whitney–Wilcoxon signed-rank test).

However, because brain drainage was reduced before CSD in older mice, they showed the most pronounced suppression of FITCD distribution in the brain and its accumulation in dcLNs than young and middle-age mice. Indeed, in 24-month-old mice, the intensity signal from FITCD was 9.5 (*p* < 0.001) and 3.7 (*p* < 0.05) times lowers in ventral part of the brain vs. with 3- and 12-months old mice; 9.0 (*p* < 0.001) and 4.4 (*p* < 0.05) times lowers in dorsal part of the brain vs. with 3- and 12-months old mice; 1.9 (*p* < 0.05) and 1.5 (ns) times lowers in dcLNs vs. with 3- and 12-months old mice (ventral part of the brain: 0.22 ± 0.07 a.u. vs. 2.11 ± 0.21 a.u. between 24- and 3-month-old mice, *p* < 0.001 and 0.22 ± 0.07 a.u. vs. 0.83 ± 0.09 a.u., *p* < 0.05 between 24- and 12-month-old mice; dorsal part of the brain: 0.20 ± 0.03 a.u. vs. 1.80 ± 0.17 a.u., *p* < 0.001 between 24- and 3-month-old mice and 0.20 ± 0.03 a.u. and 0.88 ± 0.16 a.u., *p* < 0.05 between 24- and 12-month-old mice; dcLNs: 3.11 ± 0.36 a.u. vs. 6.12 ± 0.60 a.u., *p* < 0.05 between 24- and 3-month-old mice and 3.11 ± 0.36 a.u. vs. 4.73 ± 0.62 a.u., ns between 24- and 12-month-old mice; *n* = 8 in each group; the ANOVA test with the post hoc Duncan, Bonferroni test and the Mann–Whitney–Wilcoxon signed-rank test).

Note that no increase in the plasma corticosterone levels was found in any age group after ASD and CSD, which rules out the effects of stress on brain drainage. There also were no age differences in plasma corticosterone levels at 08:00 am (the control: 205.2 ± 37.1 ng/mL in 3-month-old-mice and 217.1 ± 32.5 ng/mL in 12-month-old-mice and 221.2 ± 30.8 ng/mL in 24-month-old-mice; ASD: 219.5 ± 41.0 ng/mL in 3-month-old-mice and 214.7 ± 38.2 ng/mL in 12-month-old-mice and 227.1 ± 33.5 ng/mL in 24-month-old-mice; CSD: 212.0 ± 36.2 ng/mL in 3-month-old-mice and 220.9 ± 33.7 ng/mL in 12-month-old-mice and 228.4 ± 38.6 ng/mL in 24-month-old-mice, ns between the tested groups, *n* = 8 in each group; the ANOVA test with the post hoc Duncan test).

#### Effects of PBM + sleep and PBM + wake on the brain metabolite levels in mice of different ages

**Fig. 6:**
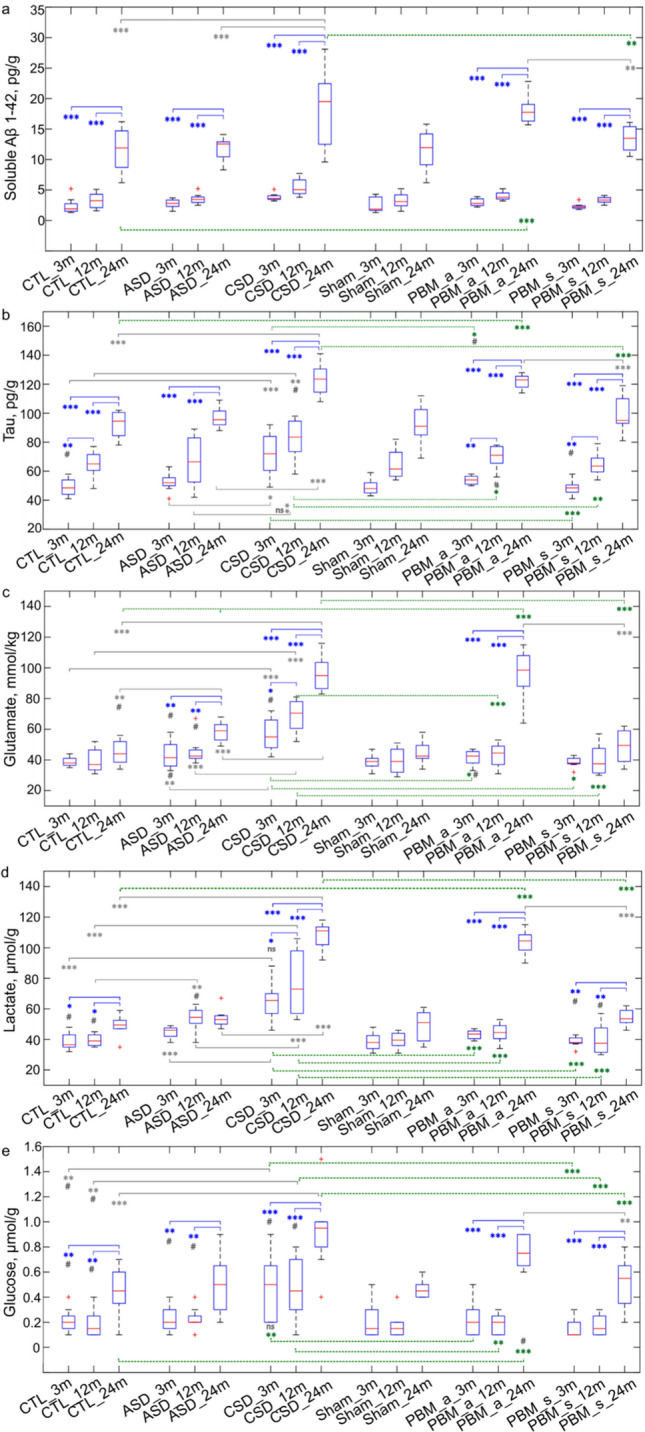
Effects of the 10-day course of PBM + sleep or PBM + wake on improvement of the brain metabolite content in ASD and CSD mice of different ages. **a–e** Quantitative analysis of the soluble brain Aβ 1–42 levels (**a**), tau protein (**b**), glutamate (**c**), lactate (**d**) and glucose (**e**) in 3-, 12-, and 24-month old mice before and after ASD and CSD as well as in 3-, 12-, and 24-month old CSD mice received the 10-day course of PBM + wake or PBM + sleep; *p < 0.05, **p < 0.01, ***p < 0.001; *n* = 8 in each group; the ANOVA test with the post hoc Duncan test (*p < 0.05, **p < 0.01, ***p < 0.001), additional pairwise comparisons by Mann–Whitney–Wilcoxon test (^#^*p* < 0.05), *n* = 8 in each group

Here, we studied the effects of ASD or CSD on the content of the typical brain metabolites in mice of different ages. Since suppression of brain drainage was detected under CSD, it was expected that a decrease in the excretion of metabolites from the brain after CSD would be found. Therefore, the possibility of maintaining normal metabolite levels in the brain of mice of different ages under CSD by receiving a course of PBM + sleep or PBM + wake was studied (Figs. 6a–e).

All subsequent comparisons in these experiments were performed using the ANOVA test with the post hoc Duncan and Bonferroni test supplemented by the Mann–Whitney–Wilcoxon signed-rank test in the case of non-Gaussian statistics or the absence of significant differences by one of the post hoc tests. The Duncan test is shown to visualize different levels of differences. The results of the Mann–Whitney–Wilcoxon test are additionally noted only in cases where the Bonferroni correction showed results at the significance threshold (Figs. 5a–e).

Figure 6a demonstrates gradual age-related increase in the soluble Aβ levels in the brain. There was a 1.7-fold (*p* < 0.05) and 6.1-fold (*p* < 0.001) an increase in the brain Aβ levels by 12 and 24 months of life vs. 3-month-old mice (3.31 ± 0.05 pg/g protein vs. 1.90 ± 0.15 pg/g protein, *p* < 0.05 between 12- and 3-month-old mice; 11.65 ± 1.25 pg/g protein vs. 1.90 ± 0.15 pg/g protein between 24- and 3-month-old mice; *n* = 8 in each group).

CSD but not ASD was associated with an additional and approximately equal increase of soluble Aβ in the brain of mice of different ages. However, these changes were statistically significant only in old mice, but not in young and middle-aged animals (18.30 ± 2.26 pg/g protein vs. 11.65 ± 1.25 pg/g, protein *p* < 0.001 between 24-month-old mice after and before CSD; 5.56 ± 0.50 pg/g protein vs. 3.31 ± 0.05 pg/g protein, not statistically significant between 12-month-old mice after and before CSD; 3.62 ± 0.12 pg/g protein vs. 1.90 ± 0.15 pg/g protein, ns between 3-month-old mice after and before CSD; *n* = 8 in each group).

Interestingly, CSD old mice treated by PBM + sleep, but not PBM + wake, demonstrated the soluble Aβ levels almost the same as its basal levels (13.40 ± 0.78 pg/g protein vs. 18.30 ± 2.26 pg/g protein, *p* < 0.01 between CSD under PBM + sleep and CSD alone; 13.40 ± 0.78 pg/g protein vs. 11.65 ± 1.25 pg/g protein, not statistically significant between PBM + sleep and the control; *n* = 8 in each group). Since CSD caused weak changes in Aβ levels in young and middle-aged mice, both PBM + sleep and PBM + wake did not significantly change its content in the brain of CSD mice, and the Aβ brain level in these mice were similar to the controls.

With regard to tau protein, similar changes were observed as with a Aβ (Fig. 5b). Indeed, a gradual increase in tau protein in the brain was 1.3 times (*p* < 0.01) by 12 months and by 1.9 times (*p* < 0.001) by 24-months (64.93 ± 3.22 pg/g protein vs. 49.12 ± 2.14 pg/g protein, *p* < 0.01 between 12- and 3-month-old mice; 92.40 ± 3.41 pg/g protein vs. 49.12 ± 2.14 pg/g protein, *p* < 0.001 between 24- and 3-month-old mice; 92.40 ± 3.41 pg/g protein vs. 64.93 ± 3.22 pg/g protein, *p* < 0.001 between 24- and 12-month-old mice; *n* = 8 in each group).

CSD but not ASD caused an increase in the brain tau level in all mice, with the most pronounced changes in older animals (123.35 ± 2.26 pg/g protein vs. 92.40 ± 3.41 pg/g protein *p* < 0.001 between 24-month-old mice after and before CSD; 82.41 ± 4.94 pg/g protein vs. 64.93 ± 3.22 pg/g protein, *p* < 0.01 between 12-month-old mice after and before CSD; 71.84 ± 5.25 pg/g protein vs. 49.12 ± 2.14 pg/g protein, *p* < 0.001 between 3-month-old mice after and before CSD; *n* = 8 in each group).

In CSD mice, PBM + wake inhibited the CSD-mediated increase in the brain tau level in young and middle-aged mice, but not in old animals. Indeed, in CSD mice aged 3- and 12 months, the brain tau level was significantly lower in the groups received PBM + wake compared to CSD alone and was similar to the basal unit of this metabolite (53.93 ± 1.11 pg/g protein vs. 71.84 ± 5.25 pg/g protein, *p* < 0.05, between 3-month-old CSD mice received and not PBM + wake; 53.93 ± 1.11 pg/g protein vs. 49.12 ± 2.14 pg/g protein, not statistically significant, between 3-month-old CSD mice received PBM + wake and the control; 70.11 ± 2.70 pg/g protein vs. 82.41 ± 4.94 pg/g protein, *p* < 0.05, between 12-month-old CSD mice received and not PBM + wake; 70.11 ± 2.70 pg/g protein vs. 64.93 ± 3.22 pg/g protein, not statistically significant, between 12-month-old CSD mice received PBM + wake and the control; *n* = 8 in each group).

In contrast, in CSD 24-month-old mice, PBM + wake did not affect the brain tau level, therefore it remained high compared to the control (121.94 ± 1.25 pg/g protein vs. 123.35 ± 2.26 pg/g protein, not statistically significant, between 24-month-old CSD mice received and not PBM + wake; 121.94 ± 1.25 pg/g protein vs. 92.40 ± 3.41 pg/g protein, *p* < 0.001, between 24-month-old CSD mice received PBM + wake and the control; *n* = 8 in each group).

However, PBM + sleep effectively kept the brain tau level within basal values in CSD mice of all ages, including old mice (48.50 ± 1.80 pg/g protein vs. 49.12 ± 2.14 pg/g protein, *p* < 0.05, not statistically significant between 3-month-old CSD mice received PBM + wake and the control; 64.62 ± 2.74 pg/g protein vs. 64.93 ± 3.22 pg/g protein, not statistically significant between 12-month-old CSD mice received PBM + wake and the control; 99.51 ± 4.48 pg/g protein vs. 92.40 ± 3.41 pg/g protein, not statistically significant, between 24-month-old CSD mice received PBM + wake and the control; *n* = 8 in each group).

The brain glutamate levels tended to increase in old mice compared to young and middle-aged mice, but these changes were not statistically significant (39.63 ± 2.75 mmol/kg vs. 38.85 ± 1.08 mmol/kg, ns between 12-and 3-month-old mice; 44.90 ± 2.82 mmol/kg vs. 38.85 ± 1.08 mmol/kg, ns between 24-and 3-month-old mice; 44.90 ± 2.82 mmol/kg vs. 39.63 ± 2.75 mmol/kg, ns between 24-and 12-month-old mice; *n* = 8 in each group).

ASD resulted in a significant increase in the brain glutamate levels only in 24-month-old mice, but not in 3- and 12-month-old animals (58.44 ± 2.29 mmol/kg vs. 44.90 ± 2.82 mmol/kg, *p* < 0.01 between 24-month-old mice after and before ASD; 43.31 ± 3.12 mmol/kg vs. 38.85 ± 1.08 mmol/kg, not statistically significant between 3-month-old mice after and before ASD; 42.62 ± 1.23 mmol/kg vs. 39.63 ± 2.75 mmol/kg, not statistically significant between 12-month-old mice after and before ASD; *n* = 8 in each group).

However, CSD caused significant increases in brain glutamate levels in mice of all ages (56.55 ± 3.77 mmol/kg vs. 38.85 ± 1.08 mmol/kg, *p* < 0.001 between 3-month-old mice after and before CSD; 68.91 ± 3.77 mmol/kg vs. 39.63 ± 2.75 mmol/kg, *p* < 0.001 between 12-month-old mice after and before CSD; 96.11 ± 3.97 mmol/kg vs. 44.90 ± 2.82 mmol/kg, *p* < 0.001 between 24-month-old mice after and before CSD; *n* = 8 in each group).

In young and middle-aged mice, both courses of PBM + sleep and PBM + wake effectively inhibited the increase in the brain glutamate levels induced by CSD, resulting in the brain glutamate levels within normal units in these groups (41.50 ± 1.71 mmol/kg vs. 38.85 ± 1.08 mmol/kg, not statistically significant between CSD 3-month-old mice received PBM + wake and the control; 39.04 ± 0.87 mmol/kg vs. 38.85 ± 1.08 mmol/kg, not statistically significant between CSD 3-month-old mice received PBM + sleep and the control; 43.12 ± 2.75 mmol/kg vs. 39.63 ± 2.75 mmol/kg, not statistically significant between CSD 12-month-old mice received PBM + wake and the control; 40.17 ± 3.53 mmol/kg vs. 39.63 ± 2.75 mmol/kg, not statistically significant between CSD 12-month-old mice received PBM + sleep and the control; *n* = 8 in each group).

In old mice, only the course of PBM + sleep, but not PBM + wake, exerted inhibitory effects on the brain glutamate content (48.92 ± 3.88 mmol/kg vs. 58.44 ± 2.29 mmol/kg between CSD 24-month-old mice received PBM + sleep and the control, not statistically significant; 96.00 ± 5.77 mmol/kg vs. 58.44 ± 2.29 mmol/kg, *p* < 0.001 between CSD 24-month-old mice received PBM + wake and the control; *n* = 8 in each group).

The same age-related differences as for the other metabolites were observed for the brain lactate content (Fig. 6d). Thus, the brain lactate level increased significantly in old mice compared to young (by 1.3 times, *p* < 0.05) and middle-aged (by 1.3 times, *p* < 0.05) mice, between which the brain values for this metabolite did not differ (51.02 ± 9.88 µmol/g vs. 38.60 ± 1.94 µmol/g, *p* < 0.05 between 24- and 3-month-old mice; 51.02 ± 9.88 µmol/g vs. 39.51 ± 1.36 µmol/g, *p* < 0.05 between 24- and 12-month-old mice; 38.60 ± 1.94 µmol/g vs. 39.51 ± 1.36 µmol/g, not statistically significant between 3- and 12-month-old mice; *n* = 8 in each group).

CSD, but not ASD was associated with significant increases in the brain lactate levels in mice of all ages (64.93 ± 4.40 µmol/g vs. 38.60 ± 1.94 µmol/g, *p* < 0.001 between 3-month-old mice after and before CSD; 76.97 ± 7.91 µmol/g vs. 39.51 ± 1.36 µmol/g, *p* < 0.001 between 12-month-old mice after and before CSD; 109.93 ± 3.23 µmol/g vs. 51.02 ± 9.88 µmol/g, *p* < 0.001 between 24-month-old mice after and before CSD; *n* = 8 in each group).

A course of PBM + wake effectively maintained the brain lactate levels within basal values in young and middle-aged sleep-deprived mice (43.10 ± 1.06 µmol/g vs. 38.60 ± 1.94 µmol/g, not statistically significant between CSD 3-month-old mice received PBM + wake and the control; 44.42 ± 2.20 µmol/g vs. 39.51 ± 1.36 µmol/g, not statistically significant between CSD 12-month-old mice received PBM + wake and the control; *n* = 8 in each group).

In old mice, a course of PBM + wake did not inhibit the sleep deprivation-induced increase in the brain lactate levels (103.50 ± 2.85 µmol/g vs. 51.02 ± 9.88 µmol/g, *p* < 0.001 between CSD 24-month-old mice received PBM + wake and the control; *n* = 8 in each group).

Importantly, during CSD, the course of PBM + sleep had an effective effect on maintaining the basal brain levels of lactate in mice of all ages, including old mice (39.40 ± 2.28 µmol/g vs. 38.60 ± 1.94 µmol/g, not statistically significant between CSD 3-month-old mice received PBM + sleep and the control; 40.00 ± 3.53 µmol/g vs. 39.51 ± 1.36 µmol/g, not statistically significant between CSD 12-month-old mice received PBM + sleep and the control; 54.41 ± 1.95 µmol/g vs. 51.02 ± 9.88 µmol/g, not statistically significant between CSD 24-month-old mice received PBM + sleep and the control; *n* = 8 in each group).

Like other metabolites, the glucose levels increased with age and were high compared with young (by 2.65 times, *p* < 0.01) and middle-aged (by 2.65 times, *p* < 0.01) mice, between which there was no difference (0.53 ± 0.07 µmol/g vs. 0.20 ± 0.03 µmol/g, *p* < 0.01 between 24- and 3-month-old mice; 0.53 ± 0.07 µmol/g vs. 0.20 ± 0.04 µmol/g, *p* < 0.01 between 24- and 12-month-old mice; 0.20 ± 0.03 µmol/g vs. 0.20 ± 0.04 µmol/g, ns between 3- and 12-month-old mice; *n* = 8 in each group) (Fig. 6e).

CSD, but not ASD significantly increased the brain glucose levels in mice of different ages (0.55 ± 0.09 µmol/g vs. 0.20 ± 0.03 µmol/g, *p* < 0.01 between 3-month-old mice after and before CSD; 0.58 ± 0.09 µmol/g vs. 0.20 ± 0.04 µmol/g, *p* < 0.01 between 12-month-old mice after and before CSD; 0.90 ± 0.05 µmol/g vs. 0.53 ± 0.07 µmol/g, *p* < 0.001 between 24-month-old mice after and before CSD; *n* = 8 in each group).

The course of PBM + wake effectively maintained the brain glucose levels within normal units in deprived mice of young and middle age, but not in old animals (0.20 ± 0.05 µmol/g vs. 0.20 ± 0.03 µmol/g, not statistically significant between CSD 3-month-old mice received PBM + wake and the control; 0.20 ± 0.03 µmol/g vs. 0.20 ± 0.04 µmol/g, not statistically significant between CSD 12-month-old mice received PBM + wake and the control; 0.80 ± 0.05 µmol/g vs. 0.53 ± 0.07 µmol/g, *p* < 0.001 between CSD 24-month-old mice received PBM + wake and the control; *n* = 8 in each group).

However, a course of PBM + sleep effectively maintained the brain glucose levels in deprived mice of all ages, including old animals (0.20 ± 0.03 µmol/g vs. 0.20 ± 0.03 µmol/g, not statistically significant between CSD 3-month-old mice received PBM + sleep and the control; 0.20 ± 0.03 µmol/g vs. 0.20 ± 0.04 µmol/g, not statistically significant between CSD 12-month-old mice received PBM + sleep and the control; 0.51 ± 0.08 µmol/g vs. 0.53 ± 0.07 µmol/g, not statistically significant between CSD 24-month-old mice received PBM + sleep and the control; *n* = 8 in each group).

It should be noted that in all groups of tested metabolites, no differences were observed between the control and sham groups (Aβ in 3-month-old mice: 1.90 ± 0.15 pg/g protein in the control and 2.04 ± 0.11 pg/g protein in the sham group, ns; Aβ in 12-month-old mice: 3.31 ± 0.05 pg/g protein in the control and 3.26 ± 0.03 pg/g protein in the sham group, ns; Aβ in 24-month-old mice: 11.65 ± 1.25 pg/g protein in the control and 11.58 ± 1.06 pg/g protein in the sham group, ns; tau protein in 3-month-old mice: 49.12 ± 2.14 pg/g protein in the control and 49.01 ± 1.94 pg/g protein in the sham group, ns; tau protein in 12-month-old mice: 64.93 ± 3.22 pg/g protein in the control and 64.80 ± 3.15 pg/g protein in the sham group, ns; tau protein in 24-month-old mice: 92.40 ± 3.41 pg/g protein in the control and 92.30 ± 3.11 pg/g protein in the sham group, ns; glutamate in 3-month-old mice: 38.85 ± 1.08 mmol/kg in the control and 38.75 ± 1.02 mmol/kg in the sham group, ns; glutamate in 12-month-old mice: 39.63 ± 2.75 mmol/kg in the control and 39.50 ± 2.16 mmol/kg in the sham group, ns; glutamate in 24-month-old mice: 44.90 ± 2.85 mmol/kg in the control and 44.75 ± 2.25 mmol/kg in the sham group, ns; lactate in 3-month-old mice: 38.60 ± 1.94 µmol/g in the control and 38.50 ± 1.70 µmol/g in the sham group, ns; lactate in 12-month-old mice: 39.51 ± 1.36 µmol/g in the control and 39.50 ± 1.25 µmol/g in the sham group, ns; lactate in 24-month-old mice: 51.02 ± 9.88 µmol/g in the control and 48.88 ± 10.00 µmol/g in the sham group, ns; glucose in 3-month-old mice: 0.20 ± 0.03 µmol/g in the control and 0.21 ± 0.05 µmol/g in the sham group, ns; glucose in 12-month-old mice: 0.20 ± 0.04 µmol/g in the control and 0.18 ± 0.09 µmol/g in the sham group, ns; glucose in 24-month-old mice: 0.53 ± 0.07 µmol/g in the control and 0.46 ± 0.08 µmol/g in the sham group, ns; *n* = 8 in each group).

## Mechanisms of PBM-mediated stimulation of drainage of sleeping brain

**Fig. 7:**
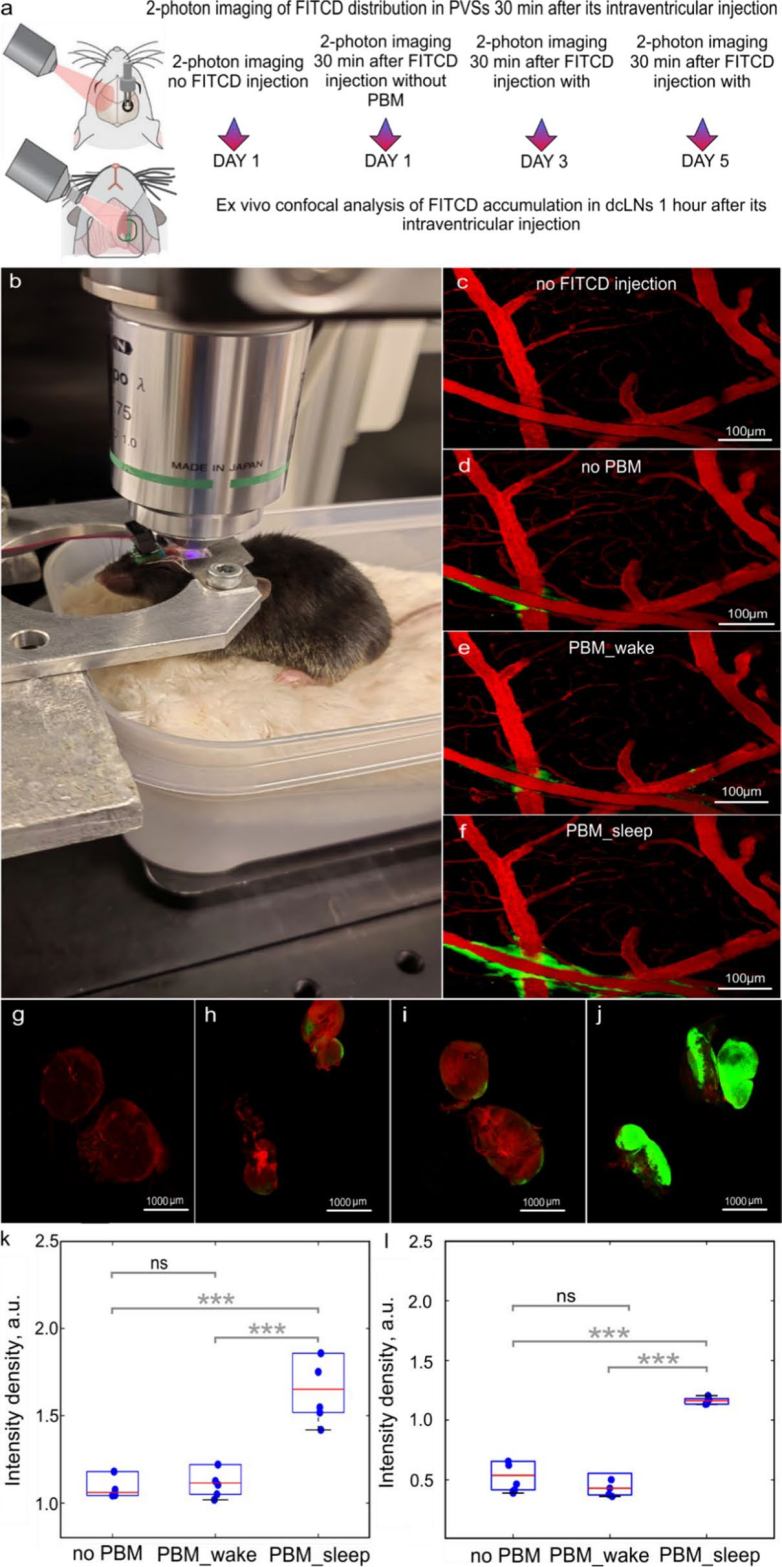
PBM effects during sleep and wake on brain drainage in 24-month-old mice. **a** Schematic illustration of the experimental design. **b** Photo showing real-time monitoring of brain drainage in a sleeping and non-anesthetized mouse using a 2-photon microscope. **c–j** Multiphoton images of the same ROI in the same old mouse without FITCD injection, 30 min after FITCD injection and without PBM (the first day of observation) as well as with PBM + sleep (the third day of the study) or with PBM + wake (the fifth day of the experiment). **k** and** l** Quantitative analysis of the signal intensity from FITCD in a.u. in PVSs (**k**) and in dcLNs (**l**); in ***p < 0.001; *n* = 5 in each group; *ns *not statistically significant, the ANOVA test with the post hoc Duncan test

To answer the question of what are the mechanisms underlying effective photostimulation of brain drainage and metabolite clearance during sleep in 24-month-old mice, real-time 2-photon imaging through optical window of the intensity of FITCD clearance from the right lateral ventricle and its distribution in the perivascular spaces (PVSs) were performed in unanesthetized mice (Fig. 7a and b). Figure 7c–f show representative images of the cerebral vessels without FITCD intraventricular injection (Fig. 7c), FITCD distribution in PVSs 30 min after tracer introduction without PBM (Fig. 7d), with PBM + sleep (Fig. 7e) or PBM + wake (Fig. 7f).

Monitoring of FITCD distribution in PVSs without PBM, during sleep (FITCD was injected via catheter at the moment of EEG recording of NREM sleep) or wakefulness was performed on the same mouse at the same ROI on the first, third and fifth days of the studies, when FITCD completely removed from PVSs. That is, exactly the same study was repeated three times on the same unanesthetized mouse. Quantitative analysis revealed minimal amounts of FITCD in PVSs without PBM, which were not different after PBM + wake, but significantly increased after PBM + sleep (1.58 ± 0.04 vs. 1.22 ± 0.05, *p* < 0.001 between the FITCD injection with PBM + sleep group and the FITCD injection without PBM group; 1.58 ± 0.04 vs. 1.24 ± 0.01, *p* < 0.001 between the FITCD injection with PBM + sleep group and the FITCD injection with PBM + wake group; 1.22 ± 0.05 vs. 1.24 ± 0.01, ns between the FITCD injection without PBM group and the FITCD injection with PBM + wake group, *n* = 5, the ANOVA test with the post hoc Duncan test) (Fig. 7k).

Additionally, in other groups using the same protocol of FITCD administration into the right lateral ventricle, ex vivo confocal analysis of the lymphatic removal of the dye from the brain into dcLNs was performed (Fig. 7a and g–j). Our ex vivo results were similar to those in vivo data and revealed a significant accumulation of FITDC in dcLNs only in the FITCD with PBM + sleep group, while in the FITCD without PBM or FITCD with PBM + wake the level of the dye was minimal and did not differ from each other (1.29 ± 0.08 vs. 0.52 ± 0.09, *p* < 0.001 between the FITCD injection with PBM + sleep group and the FITCD injection without PBM group; 1.29 ± 0.08 vs. 0.49 ± 0.07, *p* < 0.001 between the FITCD injection with PBM + sleep group and the FITCD injection with PBM + wake group; 0.49 ± 0.07 vs. 0.52 ± 0.09, ns between the FITCD injection without PBM group and the FITCD injection with PBM + wake group, *n* = 5, the ANOVA test with the post hoc Duncan test).

Thus, these results showed that PBM during sleep, but not during wakefulness, increases brain drainage, i.e., increases the movement of brain fluids through the glymphatic and/or lymphatic pathways, facilitating better removal of compounds dissolved in brain fluids to the periphery.

## Discussion

In this study, we clearly demonstrate that sleep is a “therapeutic window” for PBM in aged mice. Sleep deprivation experiments have shown that CSD, but not ASD results in significant suppression of brain drainage, accompanied by accumulation of brain metabolites such as Aβ, tau protein, glutamate, lactate, and glucose in mice of all ages. However, we observed a significant accumulation of these metabolites in the brain of old mice, which was combined with a marked suppression of brain drainage compared to young and middle-aged mice. Therefore, the changes in metabolite levels and brain drainage after CSD in 24-month-old mice were stronger than in 3- or 12-month-old mice. The decrease in metabolite clearance with age has also been found by other researchers, who clearly showed excessive deposition of Aβ and tau protein in the brain of old mice [37, 74]. One of the reasons for the impaired clearance of metabolites from the brain are morphological changes in MLVs [74]. In our previous study, we found the development of hyperplasia of MLVs leading to suppression of lymphatic drainage function in 24-month-old mice [27]. Ahn et al. also demonstrated the overgrowth of the endothelium of MLVs in old mice [74]. There is evidence that the lymphatic hyperplasia develops as compensation to the lymphatic valve dysfunction leading to a decrease in peristaltic lymphatic flow and reduction of brain drainage [75–78].

PBM is an effective method to prevent excessive accumulation of brain metabolites caused by CSD. However, in aged mice, only PBM + sleep, but not PBM + wake, has effective stimulating effects on brain drainage and removal of brain metabolites. In contrast to aged mice, in young and middle-aged animals, both PBM + sleep and PBM + wake are effective in maintaining brain metabolite levels within normal units. The obtained results are consistent with our previous data that age is a limiting factor in the effective use of PBM to stimulate brain drainage and improve its cognitive functions [27]. We clearly showed that effective photo-effect on brain drainage requires preservation of the MLV functions [27].

Here, we confirmed the hypothesis that sleep can enhance the therapeutic effects of PBM in old animals. Indeed, the course of PBM + sleep but not PBM + wake effectively reduces the level of Aβ in the brain and its meninges, which is accompanied by an improvement in cognitive function. Furthermore, only PBM + sleep exerted effective therapeutic effects in maintaining normal brain metabolite levels in chronically deprived mice.

How PBM can improve cognitive function remains poorly understood. Emerging evidence indicates the important roles of brain drainage in the resistant to neurocognitive disorders [37, 61, 79–84]. Indeed, dysfunction of MLVs results in less drainage of brain fluids to the cervical lymph nodes [37, 38, 40, 85–88]. Such disruption of MLVs also results in cognitive impairment and behavioral alterations [37]. Increasing lymphangiogenesis of MLVs via administration of the vascular endothelial growth factor improves the drainage of macromolecules to the cervical lymph nodes of elderly mice [81, 89]. Finally, disruption of MLVs worsens mouse models of AD [37]. Collectively, these findings suggest that dysfunction of brain drainage might provide an important contribution to age-related cognitive decline and neuro-degenerative disease [37, 74, 80, 81, 89, 90].

The natural activation of brain drainage during sleep [44, 45] may explain the “opening” of the therapeutic window for PBM in the aging brain. Our studies have shown that brain drainage decreases with age, which is consistent with the data of other researchers [37, 74]. Nevertheless, drainage is preserved in old mice, possibly during sleep due to the natural processes of activation of removal of metabolites from the central nervous system with the brain fluid flow.

The results of in vivo and ex vivo studies of the PBM effects during sleep and wakefulness in 24-month-old mice clearly showed that only PBM + sleep, but not PBM + wake, exerts stimulating effects on perivascular and lymphatic drainage of brain fluid in old animals.

At present, due to technical limitations in studying fluid movement in the mouse brain, it is difficult to answer the question of what mechanisms (glymphatic, lymphatic, or other) underlie the PBM-stimulating effects on brain drainage. On the one hand, there are data clearly indicating that during deep sleep, brain drainage increases due to the expansion of the size of PVSs and changes in the volume of astrocytes, which creates special spaces for the movement of brain fluids [44, 45, 91]. However, for the targeted movement of fluids in the brain, a system of special vessels must exist that will direct this movement in a certain direction. Despite the 100-year history of studying brain drainage and the known pathways for this, such as PVSs [92–96], the lymphatic vessels in the ethmoid bone [92, 97] and along the exit of the nerves from the brain [92, 97], as well as the obvious fact that the cervical lymph nodes are the first anatomical collection station for CSF from the brain [37, 74, 98], the mechanisms of metabolite removal from the central nervous system remain unclear. The proposed glymphatic hypothesis, in which the aquaporin channels play an important role in a driving force and arterial pulsation [99–101], nevertheless contains many unresolved critical physiologic issues that are actively discussed by nephrophysiologists [92, 102–106]. In addition, the fact of glymphatic flow has not been experimentally proven. A similar situation is with the lymphatic vessels, the existence of which in the brain has also not been proven [107–110]. There are original results that demonstrate circadian changes in the activity of the glymphatic and lymphatic systems of the brain [111]. However, most likely, these two drainage mechanisms work inseparably, since the obvious and indisputable facts remain that brain fluids move along PVSs and are excreted into the peripheral lymphatic system. It is important to note that the movement of tracers in the brain directly depends on their nature. Therefore, results studying brain drainage using neutral dyes or only one type of protein, such as beta-amyloid, may not provide a complete picture explaining the mechanisms of brain drainage, especially the pathways for removing metabolites from the central nervous system. Until the cerebral lymphatic vessels or glymphatic fluid flow are discovered, this question will not be answered unambiguously.

Our results clearly showed that PBM during sleep, but not during wakefulness in old mice, stimulates both fluid flow in PVSs and lymphatic drainage. In our earlier studies [27], morphological changes in MLVs were revealed in 24-month-old mice, which was accompanied by a decrease in brain drainage, including lymphatic drainage, which was also shown by other researchers [37, 74]. These facts suggest that PBM during sleep is effective in old mice due to stimulation of brain fluid flow, including perivascular and lymphatic pathways.

In addition, the decrease in PBM efficiency in 24-month-old mice may be related to morphological changes in the skull that reduce its ability to transmit light [112]. Indeed, the transmission analysis revealed that the transmittance spectra for 1050 nm light were 60 ± 5%, 48 ± 5%, and 40 ± 5% for 3-, 12-, and 24-month-old mice. Thus, it could be expected that by increasing the dose of PBM for 24-month-old mice by 1.5 times and for 12-month-old mice by 1.25 times compared to 3-month-old mice, the efficacy of PBM could be increased, which requires further detailed studies. However, in this study, in order to maintain standardization in the study and taking into account the goal of studying the enhancement of the efficacy of PBM during sleep using the same dose for all ages, this task was not solved. In addition, considering the aging MLVs [74], which plays a key role in brain drainage and the removal of metabolites from its tissues, it is possible that simply increasing the dose of PBM will not provide the desired increase in efficacy. For example, as was observed with the effects of PBM on increasing the permeability of the blood–brain barrier [113]. In this our previous study, we proved that increasing the dose of PBM is not a decisive factor in achieving effective PBM effects.

## Conclusion

In sum, our results revealed that sleep is a therapeutic window for PBM. Indeed, the decrease in brain drainage with age is accompanied by the accumulation of metabolites in its tissues leading to a toxic effect on neurons and cognitive dysfunction. CSD exacerbates accumulation of metabolites in the brain, and more significantly in oldy mice than in young and middle-aged animals. However, old mice, unlike young and middle-aged mice, are insensitive to PBM + wake, which is not effective in stimulation of brain drainage and removing metabolites from its tissues. Importantly, in old mice, only PBM + sleep increases resistance to CSD by maintaining brain metabolites at the basal levels through stimulation of drainage, which is associated with improved cognitive function. Thus, PBM + sleep opens up new possibilities for the effective use of phototherapy for diseases of the aging brain as well as for maintaining active longevity.

## Data Availability

The data that support the findings of this study are available on request from the corresponding author.
